# Transcriptional repression by a secondary DNA binding surface of DNA topoisomerase I safeguards against hypertranscription

**DOI:** 10.1038/s41467-023-42078-9

**Published:** 2023-10-13

**Authors:** Mei Sheng Lau, Zhenhua Hu, Xiaodan Zhao, Yaw Sing Tan, Jinyue Liu, Hua Huang, Clarisse Jingyi Yeo, Hwei Fen Leong, Oleg V. Grinchuk, Justin Kaixuan Chan, Jie Yan, Wee-Wei Tee

**Affiliations:** 1 Institute of Molecular and Cell Biology (IMCB), Agency for Science, Technology and Research (A*STAR), 61 Biopolis Drive, Proteos, Singapore, 138673 Republic of Singapore; 2 Department of Obstetrics and Gynecology, Guangdong Provincial Key Laboratory of Major Obstetric Diseases, Guangzhou, China; 3 Guangdong Provincial Clinical Research Center for Obstetrics and Gynecology, Guangzhou, China; 4 Guangdong-Hong Kong-Macao Greater Bay Area Higher Education Joint Laboratory of Maternal-Fetal Medicine, Guangzhou, China; 5 The Third Affiliated Hospital of Guangzhou Medical University, Guangzhou, China; 6 Department of Physics, National University of Singapore, Singapore, 117551 Singapore; 7 Centre for Bioimaging Sciences, National University of Singapore, Singapore, 117557 Singapore; 8 Bioinformatics Institute (BII), A*STAR, 30 Biopolis Street, Matrix, Singapore, 138671 Singapore; 9 Genome Institute of Singapore (GIS), A*STAR, 60 Biopolis Street, Genome, Singapore, 138672 Singapore; 10 Department of Physiology, Yong Loo Lin School of Medicine, National University of Singapore, Singapore, Singapore; 11 Electrophysiology Core Facility, Yong Loo Lin School of Medicine, National University of Singapore, Singapore, Singapore; 12 Mechanobiology Institute, National University of Singapore, Singapore, 117411 Singapore; 13 NUS Centre for Cancer Research, Yong Loo Lin School of Medicine, National University of Singapore, Singapore, Singapore

**Keywords:** Transcription, Enzyme mechanisms

## Abstract

Regulation of global transcription output is important for normal development and disease, but little is known about the mechanisms involved. DNA topoisomerase I (TOP1) is an enzyme well-known for its role in relieving DNA supercoils for enabling transcription. Here, we report a non-enzymatic function of TOP1 that downregulates RNA synthesis. This function is dependent on specific DNA-interacting residues located on a conserved protein surface. A loss-of-function knock-in mutation on this surface, R548Q, is sufficient to cause hypertranscription and alter differentiation outcomes in mouse embryonic stem cells (mESCs). Hypertranscription in mESCs is accompanied by reduced TOP1 chromatin binding and change in genomic supercoiling. Notably, the mutation does not impact TOP1 enzymatic activity; rather, it diminishes TOP1-DNA binding and formation of compact protein-DNA structures. Thus, TOP1 exhibits opposing influences on transcription through distinct activities which are likely to be coordinated. This highlights TOP1 as a safeguard of appropriate total transcription levels in cells.

## Abstract (web summary)

Aberrant hypertranscription in cells can affect development and disease. Here, the authors show that DNA topoisomerase I prevents hypertranscription; not through its catalytic function, but through a DNA binding mechanism.

## Introduction

The absolute amount of mRNA in a cell is increasingly recognized as an important biological property of cells^1–4^. In contrast to changes in gene expression patterns where subsets of genes are upregulated or downregulated, modulation in transcriptional output changes the absolute amount of RNA production without significant perturbation to existing transcriptomic signatures.

Timely elevation in total transcriptional output, also known as global transcriptional amplification or hypertranscription, is critical for many normal developmental processes, including embryonic development, stem cell amplification, adult organ maintenance and tissue regeneration^1,2,5,6^. In the same vein, precocious increase in transcriptional output could cause disease, as is the case for MYC-driven cancers and Down syndrome-associated leukaemia, and was recently found in many cancers to associate with poor prognosis^3,4,7–9^. Hitherto, only few protein regulators of the process were identified and molecularly investigated in detailed, specifically c-Myc and Chd1 in the context of cancer and early embryonic development, respectively, and both are factors that initiate hypertranscription^7,10–12^. Much less is known about factors that prevent hypertranscription, with known examples being the nucleosomes and cyclin D1^13,14^.

In eukaryotes, DNA topoisomerase I (TOP1) is the only type 1B topoisomerase that removes helical stress in DNA by a nicking, swivelling and re-ligation mechanism^15^. This enzymatic activity is required to ensure transcription processivity by preventing the built up of inhibitory DNA supercoils^16^. However, it was observed that TOP1’s role in transcription is much more diverse than maintaining transcription elongation; it is actually involved in virtually every step of the transcription cycle from initiation, elongation, re-initiation, and even repression^17–23^. Interestingly, not all of these functions require catalytically active TOP1^24^. In fact, it was observed in vitro that catalytically dead mutant TOP1 can equally mediate transcription activation and repression^19,25,26^. This indicates that besides its enzymatic function, TOP1 has additional molecular activities that are as important for regulating transcription.

**Fig. 1:**
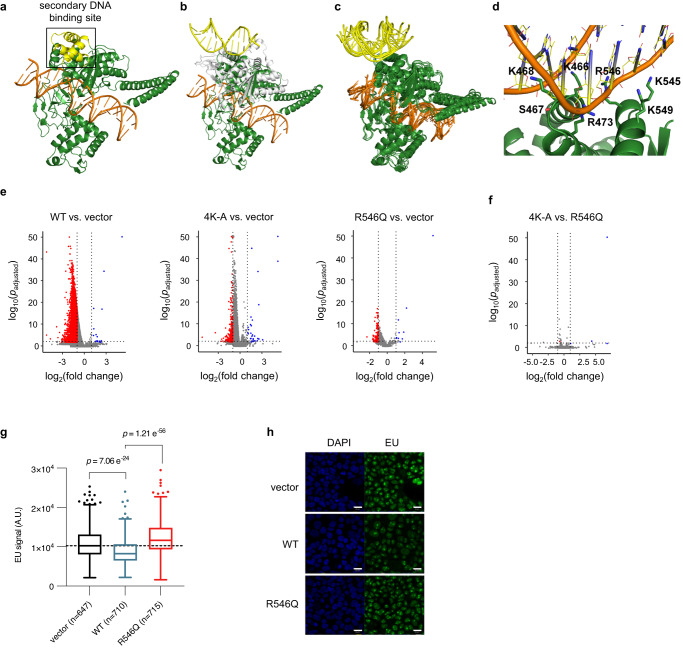
A secondary DNA binding site on TOP1 is required for transcriptional downregulation. **a** Crystal structure of human TOP1 (green) complexed with DNA (orange) at the primary DNA binding site (catalytic pocket) (PDB 1A36). The boxed region highlights the conserved surface on core subdomain III (yellow) which is the secondary DNA binding site. **b**–**d** Molecular models of human TOP1 covalent complex (TOP1cc) (TOP1 in green, DNA in orange) in complex with DNA at secondary binding site (yellow). **b** Crystal structure of human TOP1 bound to DNA at the active site (PDB 1A36) superimposed with the crystal structure of *D. radiodurans* TOP1B (white) bound to DNA at the secondary binding site (PDB 3M4A). **c** Superimposition of final MD trajectory structures of TOP1cc in complex with DNA at secondary binding site. **d** A representative structure from the MD simulations showing the binding interface between the secondary binding site and DNA; the DNA-interacting residues are shown as sticks. **e**, **f** Volcano plots of RNA-seq analyses comparing the indicated samples. Blue and red denote down- and upregulated differentially expressed genes (DEGs), as determined using DESeq2 (see details in ‘Methods’). **g** Boxplots of EU signal in single cells. Centre lines represent median, box limits represent upper and lower quartiles, whiskers represent 1.5× interquartile range, points represent outliers. *n* = 647 (vector), *n* = 710 (WT), *n* = 715 (R546Q) single-cell quantifications. Mann–Whitney test was performed; *p* values (two-tailed) are indicated above the comparisons. Source data are provided as a Source data file. **h** Representative images of EU labelling of cells from confocal microscopy imaging. Scale bars: 20 µm.

Aside from binding DNA in its catalytic pocket, TOP1 also possesses a secondary DNA binding site located on a conserved surface of the protein^27–31^ (Fig. 1a). Binding of DNA at this secondary site has been associated with the induction of DNA conformational changes and recognition of DNA topology^30,31^. This is thought to occur through TOP1 binding at DNA crossovers, where its two DNA binding sites bind simultaneously to the two juxtaposed segments of DNA^29^. However, the functional significance of this secondary DNA binding site and its role in various DNA conformations in vivo are not yet understood. Given the involvement of DNA topological and structural changes in the transcription process, it is plausible that the secondary DNA binding site of TOP1 plays an important role in the regulation of transcription. To investigate this, we employed structural modelling, mutagenesis in cells, and biophysical assays to explore the functional relevance of the secondary DNA binding surface in transcription.

We discovered that the secondary DNA binding surface of TOP1 is required for transcriptional downregulation, which is necessary to prevent aberrant hypertranscription in mESCs. The elevated transcription in mutant mESCs is accompanied by reduced TOP1 occupancy on chromatin and change in genomic supercoiling levels. We demonstrated that mutation on the secondary DNA binding site does not alter TOP1 catalytic activity. Instead, mutant TOP1 is compromised in promoting DNA conformational changes associated with DNA looping, and in stabilizing the binding-dependent DNA conformation. Finally, mutant mESCs with elevated levels of global transcription responded differently to neuronal differentiation protocols compared to wild-type cells, indicating that the molecular changes have physiological implications.

Therefore, our findings highlighted an under-appreciated repressive role of TOP1 in transcriptional control. We propose that the TOP1 binding to DNA through its secondary DNA binding site exerts a critical function in restraining transcription, which ensures appropriate transcriptional levels are maintained in cells.

## Results

### Secondary DNA binding site on TOP1 is required for transcriptional downregulation

To enable mutational studies in cells, we first identified candidate DNA-interacting residues on the secondary DNA binding site by performing molecular dynamics (MD) simulations. We first obtained an initial model of human TOP1 bound to DNA at both the active and secondary binding sites (Fig. 1b) by structural alignment of the secondary DNA binding motifs of human TOP1 with a bacterial TOP1 structure that is bound to a DNA duplex at its secondary binding site^30^, and simulated it in four replicate MD simulation runs. Although there were initially atomic clashes between human TOP1 and the secondary DNA due to low sequence similarity between human TOP1 and *Deinococcus radiodurans* TOP1B at the secondary DNA binding interface, we were able to identify a simulation run in which the secondary DNA remained stably bound to TOP1. Next, using the final trajectory structure from this stable run as the initial structure, four replicate runs with reassigned atomic velocities were performed. The secondary DNA remained bound to TOP1 in a similar manner in all four simulation runs, with one end tilting towards the active site DNA (Fig. 1c), thus representing a stable model of human TOP1 bound to DNA at both the active and secondary binding sites. This is consistent with this surface being a bona fide DNA binding site. Upon examining the secondary TOP1–DNA binding interface, we identified seven amino acid residues involved in hydrogen bonding with the phosphate backbone, deoxyribose moieties, and nitrogenous bases of the bound DNA (Fig. 1d and Table 1). Notably, these include K466, K468, K545 and K549, which were previously predicted to interact with DNA and separately shown to confer specificity for binding to supercoiled DNA substrate^29,31^. The other three residues, S467, R473 and R546, are newly identified, and surprisingly form even more persistent hydrogen bonds with DNA than the lysine residues (Table 1). These three novel DNA-interacting residues, like the four lysine residues, are also highly conserved in type 1B topoisomerases from different species (Supplementary Fig. 1a). Furthermore, R546 has a disease-associated mutation R546Q^32,33^, which further lends support to our hypothesis that these DNA-interacting residues could be functionally important.

**Table 1:** Hydrogen bonds between TOP1 and secondary DNA

TOP1 residue	DNA nucleotide atom^a^	% of time bond is present
K466	Thy116 OP1	44.45
	Cyt115 O3'	12.73
S467	Thy117 OP1	91.57
K468	Thy116 O3'	73.60
	Thy116 O2	28.80
	Thy117 OP1	25.80
	Thy117 O2	21.92
	Ade7 N3	11.00
	Gua8 O4'	7.55
R473	Gua10 OP1	98.88
	Gua9 O3'	82.35
R546	Cyt11 OP1	94.43
K545	Gua12 OP2	18.70
	Gua12 OP1	8.48
K549	Cyt11 OP2	58.48
	Cyt11 O5'	5.23

^a^DNA nucleotides follow the numbering in the PDB structure 3M4A.

To determine if the identified DNA-interacting residues could disrupt DNA binding at the secondary binding site if mutated, we introduced the quadruple K466A-K468A-K545A-K549A mutations into the stable structure of TOP1 complexed with secondary DNA. This mutated structure was then simulated for 200 ns in four replicate runs. The secondary DNA rapidly unbound from the secondary site in three of the runs (after about 10, 10, and 40 ns, respectively) and remained loosely bound to TOP1 at the secondary site in the fourth run (Supplementary Fig. 1b), substantiating the requirement for the lysine residues in binding DNA. We similarly evaluated the impact of the R546Q mutation in MD simulations. The secondary DNA unbound from TOP1 after 45 ns in one run and was unstable in another run, rotating around the binding site during the simulation (Supplementary Fig. 1c). While it remained bound in the other two runs, the binding free energy is less favourable compared to that in the wild-type complex (Supplementary Fig. 1d). Taken together, the modelling data show that mutation of DNA-interacting residues on the secondary surface likely affects DNA binding.

Therefore, we generated constructs for overexpression of TOP1 variants with mutations of selected DNA-interacting residues to test their effects on transcription in 293T cells. Specifically, we generated the TOP1(4K-A) variant where the previously characterized K466, K468, K545 and K549 were each substituted with an alanine, and the TOP1(R546Q) variant which harboured the amino acid substitution R546Q. We separately overexpressed these variants and wild-type TOP1 (TOP1(WT)) in 293T cells and compared each overexpression with vector-transfected control cells. We sorted for equal numbers of cells with successful transfection, verified that TOP1 variant proteins were expressed to similar levels as indicated by western blot (Supplementary Fig. 2a), and performed cell number-normalized RNA-seq experiment with ERCC spike-ins^34^.

Interestingly, when comparing TOP1(WT)-overexpressing cells to vector control, we observed a large number of downregulated differentially expressed genes (DEGs) in the TOP1(WT)-overexpressing cells (*n* = 7279), which was an order of magnitude larger than in TOP1(4K-A)- and TOP1(R546Q)-overexpressing cells (*n* = 296 and *n* = 132, respectively) (Fig. 1e). In contrast, the numbers of upregulated DEGs were similar in all three comparisons (*n* = 16, *n* = 31, and *n* = 11 for TOP1(WT), TOP1(4K-A) and TOP1(R546Q), respectively). Considering that all three TOP1 variant-overexpressing cells exhibited similar levels of the exogenous protein (Supplementary Fig. 2a), it appears unlikely that the widespread downregulation of mRNA in TOP1(WT)-cells is a general response to protein overexpression. This observation was supported by additional experimental findings, including immunofluorescence imaging and Western blot analyses, which showed no elevation in the DNA damage response marker γH2A.X (Supplementary Fig. 2b, c), as well as cell cycle analyses that demonstrated similar profiles between TOP1(WT) cells and vector cells (Supplementary Fig. 2d). Furthermore, gene set enrichment analyses (GSEA) performed on RNA-seq comparing TOP1(WT) cells with vector cells did not reveal any upregulation of DNA Damage Repair (DDR) and Unfolded Protein Response (UPR) pathways (Supplementary Data 1). We therefore conclude that the widespread downregulation of mRNA levels in TOP1(WT)-overexpression cells is TOP1 dependent. This effect, however, is mitigated when DNA-interacting residues on the secondary DNA binding site are mutated.

Notably, when comparing the RNA-seq profiles of TOP1(4K-A)- and TOP1(R546Q)-overexpressing cells, we observed a striking similarity (Fig. 1f), indicating that the loss of a single critical residue on this surface is sufficient to disrupt its function. As a result, we decided to focus the remainder of our studies on investigating the effects of the single R546Q mutation.

We note that RNA-seq measures RNA levels at steady states, which is the combined outcome of RNA synthesis and turnover. Given TOP1’s extensive involvement in the transcription process, we wanted to assess if the downregulation of RNA following TOP1(WT)-overexpression could be due, at least in part, to decreased transcription. To test this, we performed EU labelling of nascent transcription in TOP1(WT)- or TOP1(R546Q)-overexpressing cells and vector control cells. Indeed, EU signal is lower for TOP1(WT)-overexpressing cells relative to vector control, and to TOP1(R546Q)-overexpressing cells (Fig. 1g, h). This indicates that transcriptional decrease can indeed contribute to the RNA downregulation observed in RNA-seq for TOP1(WT)-overexpressing cells, and this transcriptional decrease is mitigated by mutations on the secondary DNA binding surface.

Finally, the transcriptional decrease in TOP(WT)-overexpressing cells is accompanied by reduction in total RNA content (Supplementary Fig. 3a). Overexpression of the catalytically dead TOP1, TOP1(Y723F), also leads to reduction in total RNA content (Supplementary Fig. 3b), indicating that the catalytic activity of TOP1 is not required for the transcriptional repression observed. Therefore, our results indicate that the secondary DNA binding surface of TOP1, including the R546 residue, is important for TOP1-mediated downregulation of RNA in cells.

### Endogenous mutation of secondary DNA binding site leads to hypertranscription in mESCs

**Fig. 2:**
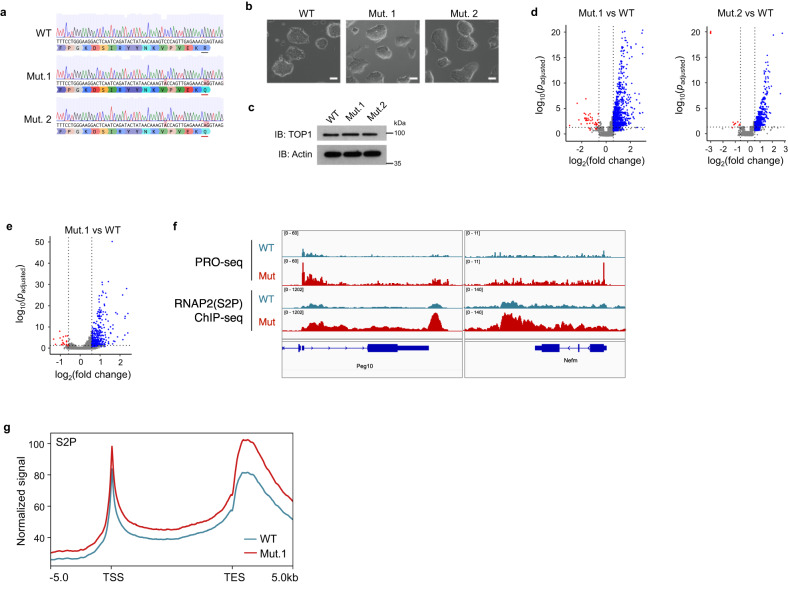
Endogenous mutation of secondary DNA binding site leads to hypertranscription in mESCs. **a** Sanger sequencing traces of a portion of exon15 of *Top1* in mutant and wildtype mESCs*;* R548Q substitutions are underlined in red. **b** Bright field images of mESC colonies. Scale bars: 20 µm. **c** Western blot of lysates obtained from equal number of cells. Actin was used as loading control. **d**, **e** Volcano plots of RNA-seq (**d**) and PRO-seq (**e**) analyses. Red and blue indicated down- and up-regulated DEGs, as determined using DESeq2 (see details in ‘Methods’). **f** Genome browser screenshot of PRO-seq and elongating RNAP2 (‘S2P’) ChIP-seq signals over representative genes. Only PRO-seq signals in the transcribing directions are shown. **g** Metaplot of ChIP-seq signals of elongating RNAP2(S2P) over all genes.

To examine the role of the secondary DNA binding site of TOP1 in a physiological setting, we generated homozygous knock-in mutation of R548Q (which is homologous to R546Q of human TOP1) in mESCs. We isolated two independent mutant clones, Mut.1 and Mut.2, and noted that they are similar to wild-type (WT) cells in terms of colony morphology and TOP1 protein levels (Fig. 2a–c).

If mutation of the secondary DNA binding site indeed leads to a loss of transcriptional repression, we would expect to see RNA upregulation in the mutant mESCs. Thus, we performed RNA-seq with cell number normalization experiments, comparing Mut.1 and Mut.2, respectively, with WT cells. The results showed that in both mutant cell lines, there is obvious bias for gene upregulation (Fig. 2d).

To ascertain if this change in RNA is attributed to changes in RNA synthesis, as was the case with 293T overexpression cells, we performed precision nuclear run-on sequencing (PRO-seq) experiments in mutant and WT cells to measure nascent RNA levels. We observed predominantly upregulated nascent transcripts in mutant compared to WT mESCs (Fig. 2e), consistent with increased transcription occurring in the mutant. The increased nascent transcription occurs over gene bodies, and we did not observe any apparent transcription readthrough at the transcription termination site, suggesting that there is increased transcription elongation but not defect in transcription termination (Fig. 2f). Additionally, chromatin immunoprecipitation followed by sequencing (ChIP-seq) for elongating RNA Polymerase II (RNAP2), which is phosphorylated on serine 2 of its C-terminus domain (S2P), revealed that elongating RNAP2 levels were also higher over genes in the TOP1 mutant cell lines, again supporting a general increased in transcription that is akin to hypertranscription (Fig. 2f, g and Supplementary Fig. 4). Therefore, loss of a DNA-interacting residue on the secondary surface of TOP1 leads to elevated transcription, indicating that the surface is otherwise required to limit transcription in mESCs.

### Mutation of secondary DNA binding site reduces TOP1 binding on chromatin

**Fig. 3:**
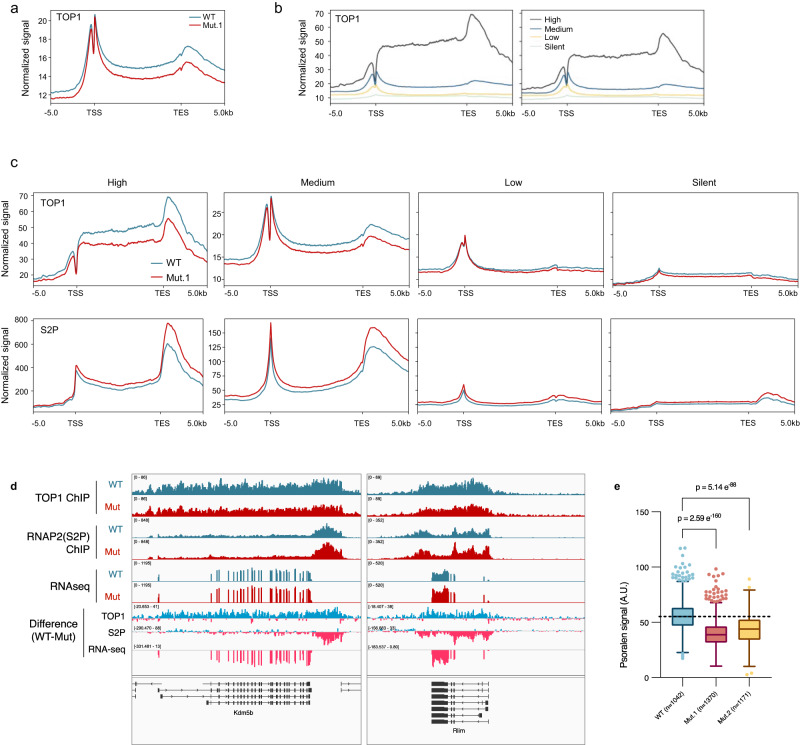
Mutation of secondary DNA binding site reduces TOP1 binding on chromatin and negative supercoiling in genome. **a**, **b** Metaplot of TOP1 ChIP-seq signals over all genes (**a**), or genes classified according to gene expression levels (**b**). **c** Metaplots of TOP1 and elongating RNAP2 (‘S2P’) ChIP-seq signal of over genes classified according to expression levels. Note the difference in y-axis for ‘High’ genes compared to the other categories. **d** Genome browser screenshots of ChIP- and RNA-seq data showing representative genes which are bound and affected by TOP1(R54Q) mutation. The ‘Difference’ tracks were obtained by subtracting mutant signal from corresponding WT signal. The turquoise and pink colours denote positive and negative values, respectively, from the subtraction. **e** Boxplots of psoralen signal in single cells. Centre lines represent median, box limits represent upper and lower quartiles, whiskers represent 1.5× interquartile range, points represent outliers. *n* = 1042 (WT), *n* = 1370 (Mut.1), *n* = 1171 (Mut.2) single-cell quantifications. Mann–Whitney test was performed; *p* values (two-tailed) are indicated above the comparisons. Source data are provided as a Source data file.

To determine how R548Q mutation in TOP1 could lead to transcriptional upregulation in mESCs, we compared TOP1 chromatin binding in mutant and WT mESCs by ChIP-seq. We found binding levels of endogenous TOP1(R548Q) to be ubiquitously lower than wild-type TOP1 (Fig. 3a and Supplementary Fig. 5a), consistent with what we observed in our MD simulations, which predicted DNA binding to be negatively impacted by R546Q (Supplementary Fig. 1c, d).

Notably, notwithstanding the lower binding of mutant TOP1 relative to wild-type TOP1, the binding levels of both proteins still positively correlate with gene expression in cells (Fig. 3b and Supplementary Fig. 5b), as was previously reported by others^20^. This suggests that the role of TOP1 in facilitating transcription is preserved in mutant TOP1 protein. However, in comparing genes of the same expression class between mutant and WT cells, mutant TOP1 binding is lower despite higher transcription, as indicated by their higher chromatin levels of elongating RNAP2 (Fig. 3c and Supplementary Fig. 5c). Some of the genes with increased transcription but lower TOP1 binding in mutant cells include developmentally important genes such as *Kdm5b* and *Rlim* (Fig. 3d). We further determined that the relative higher levels of RNAP2(S2P) on chromatin in mutant is not due to increased protein-protein interaction between mutant TOP1 with RNAP2(S2P) or its associated transcription elongation complex by co-immunoprecipitation experiment (Supplementary Fig. 5d). We also confirmed that lower mutant TOP1 occupancy on genes is not due to mis-targeting of the mutant protein to other regions of the genome, as we observed no difference in the genomic distribution of chromatin bound mutant and wild-type TOP1 (Supplementary Fig. 5e).

As transcription, TOP1 enzymatic activity, and genomic supercoiling levels are intimately linked, we wanted to know whether genomic supercoiling levels in mutant cells could be affected. Thus, we used biotinylated-trimethylpsoralen (bTMP), which preferentially intercalates into negatively supercoiled DNA, and confocal imaging to assess supercoiling levels in mutant and WT mESCs^35,36^. We observed lower negative supercoiling levels in mutant compared to WT mESCs (Fig. 3e and Supplementary Fig. 6).

In summary, we demonstrated that TOP1 mutant mESCs, which display hypertranscription, exhibit reduced TOP1 occupancy on chromatin that is accompanied by decreased levels of negative genomic supercoiling. These findings support the notion that WT TOP1 can impose a transcriptional constraint on genes when bound to chromatin.

### Mutation of secondary DNA binding site does not affect catalytic activity

Given the increased transcription in mutant mESCs, we wanted to also determine if this could be due to mutant TOP1 being more catalytically active. As such, we performed plasmid relaxation assays with purified recombinant TOP1(WT) and TOP1(R546Q) proteins.

TOP1’s relaxation of supercoiled DNA is the combined outcome of enzyme-substrate association, catalysis (which involves DNA cleavage, strand-rotation, and re-ligation), and enzyme dissociation. Therefore, to specifically compare the catalytic activity rates of TOP1(WT) and TOP1(R546Q), it is important to eliminate the influence of enzyme association/dissociation by conducting the assay in conditions where TOP1 binding is distributive^37^. It is established that distributive TOP1-DNA binding occurs in higher monovalent salt conditions, between 150-200 mM concentrations^37,38^. We first empirically verified that both TOP1(WT) and TOP1(R546Q) recombinant proteins have maximal activities in these conditions (Supplementary Fig. 7), and proceeded to carry out the plasmid relaxation assays under 200 mM salt conditions.

**Fig. 4:**
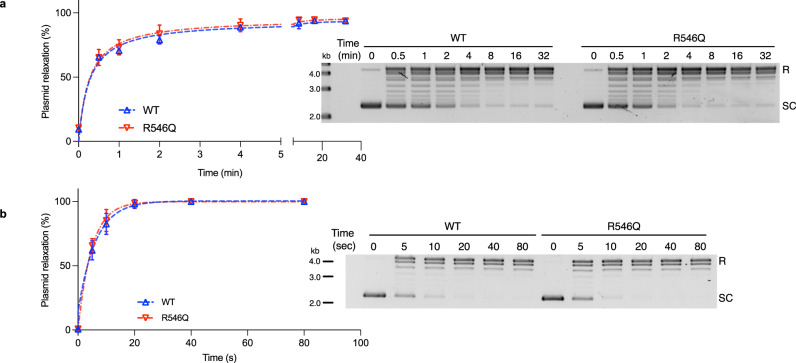
Mutation does not alter TOP1 catalytic activity. **a**, **b** Plasmid relaxation activities of TOP1(WT) and TOP1(R546Q) proteins under excess DNA (**a**) or excess enzyme (**b**) conditions. Data plotted is mean ± SD, *n* = 3 independent experiments. Inserts are representative DNA gel images of products from the assay. R relaxed plasmid, SC supercoiled plasmid. Source data are provided as a Source data file.

We conducted the assay with excess supercoiled DNA relative to enzyme, and found that the rates of plasmid relaxation by TOP1(WT) and TOP1(R546Q) proteins are nearly identical (Fig. 4a). We then similarly performed the assay with excess enzyme relative to supercoiled DNA. Again, the rates of plasmid relaxation by TOP1(WT) and TOP1(R546Q) proteins are nearly identical (Fig. 4b). The similar outcomes irrespective of enzyme/substrate ratio attests to the minimal influence of TOP1-DNA binding on plasmid relaxation in the assay conditions^39^. Therefore, we conclude that the mutation does not alter catalytic activity of TOP1. Of note, this is consistent with an earlier observation by others that showed mutations on the homologous secondary DNA binding site of *vaccinia* TOP1 to have no effect on supercoil relaxation activity^30^.

### Mutation of secondary DNA binding site impairs TOP1-DNA binding and associated DNA conformations

It was previously observed that TOP1 when incubated with DNA preferentially binds at the bases of DNA loops^27,40–42^. It is presumed that this is facilitated by its two DNA binding sites simultaneously forming interactions with distal DNA segments that are in proximity, resulting in a bridged conformation^29^. Based on our MD simulations and ChIP-seq analyses, which indicate that the R546Q mutation would disrupt TOP1-DNA binding (Fig. 3a, Supplementary Fig. 1c, d and 4), we posit that R546Q may also impact TOP1’s interaction with DNA and formation of associated DNA conformations.

**Fig. 5:**
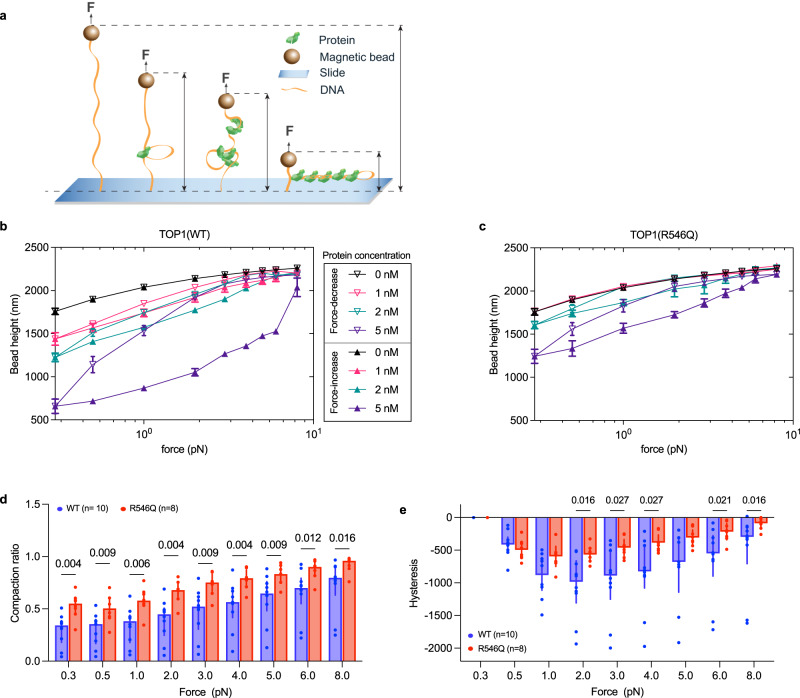
Mutation impairs TOP1’s ability to induce and stabilize binding-dependent DNA structural change. **a** Schematic representation of DNA tether held under stretching force, F, in a magnetic tweezer experiment setup. The provided illustrations showcase potential conformational changes in DNA that may occur following TOP1 protein binding. ε denotes DNA extension, which decreases as DNA is compacted by protein binding. **b**, **c** Representative force-height curves obtained from a DNA tether in a force-decrease scan (hollow symbols) followed by a force-increase scan (solid symbols) at various concentrations of TOP1(WT) (**b**) and TOP1(R546Q) (**c**). Data represented are mean ± SD of DNA extension fluctuations during the recording time window. Source data are provided as a Source Data file. **d** Compaction ratio of DNA tether when incubated with 5 nM TOP1 protein. *n* = 10 (WT), *n* = 8 (R546Q) independent DNA tethers. Bar height represent median, error bars represent interquartile range. Mann–Whitney test was used for comparing the two groups; *p* values (two-tailed) are indicated above the comparisons. Source data are provided as a Source data file. **e** Hysteresis, which is calculated by subtracting extension in force-decrease scan from extension in force-increase scan, in DNA tethers when incubated with 5 nM TOP1 protein. *n* = 10 (WT), *n* = 8 (R546Q) independent DNA tethers. Bar height represent median, error bars represent interquartile range. Mann–Whitney test was used for comparing the two groups; only *p* values (two-tailed) <0.05 are shown above the comparisons. Source data and other *p* values are provided in Source data file.

To test this, we set up magnetic tweezer experiments where DNA tethers are immobilized at one end on a cover slip and at the other end on a superparamagnetic bead. The tethered DNA is vertically extended by a magnetic force applied on the bead, and the force can be varied by manipulating the distance between a pair of magnets and the bead (Fig. 5a). DNA-protein interactions following incubation with protein can then be inferred based on shifts in the mechanical responses of single DNA tethers, including change in DNA extension length that is proxied by bead height^43,44^.

We first performed force-decrease scans (Fig. 5b, hollow symbols) followed by force-increase scans (solid symbols) with increasing concentrations of TOP1(WT) protein. We observed that TOP1(WT) at low (nanomolar) concentration is able to shorten DNA extension at sub-piconewton forces (Fig. 5b, for example, compare bead height at 2 nM protein with that of no protein control). The observed DNA ‘compaction’ by TOP1(WT) binding strongly suggests DNA looping caused by the engagement of TOP1(WT) with two distal DNA segments.

Next, we performed force scanning analysis for TOP1(R546Q). In comparison to TOP1(WT), TOP1(R546Q) is apparently impaired in its ability to cause DNA compaction (Fig. 5c). To better compare their differences, we defined a compaction ratio, which was calculated by dividing the bead height during the force-increase scan in the presence of 5 nM TOP1 protein by the bead height of naked DNA at the corresponding force. Our results showed that the compaction ratio for TOP1(WT) was significantly lower than that for TOP1(R546Q) at each force (Fig. 5d). This indicates that the mutation impaired TOP1’s ability to mediate DNA compaction, suggesting that the mutation site is otherwise involved in TOP1 binding and organizing DNA into bridged conformations. We further determined the resistance of the compacted DNA formed with 5 nM TOP1(WT) or TOP1(R546Q) to forces by calculating the hysteresis between forward and reverse force scans (bead height during force-increase minus that during force-decrease scans). As shown in Fig. 5e, hysteresis for TOP1(R546Q) is diminished compared to TOP1(WT). This indicates that the resultant compact DNA conformation is less stable with TOP1(R546Q) than with TOP1(WT), consistent with defective DNA binding with the R546Q mutation. Collectively, these results indicate that the secondary DNA binding site, which includes R546, plays a crucial role in DNA binding and associated DNA conformation changes.

### Mutant TOP1 mESCs showed altered differentiation

**Fig. 6:**
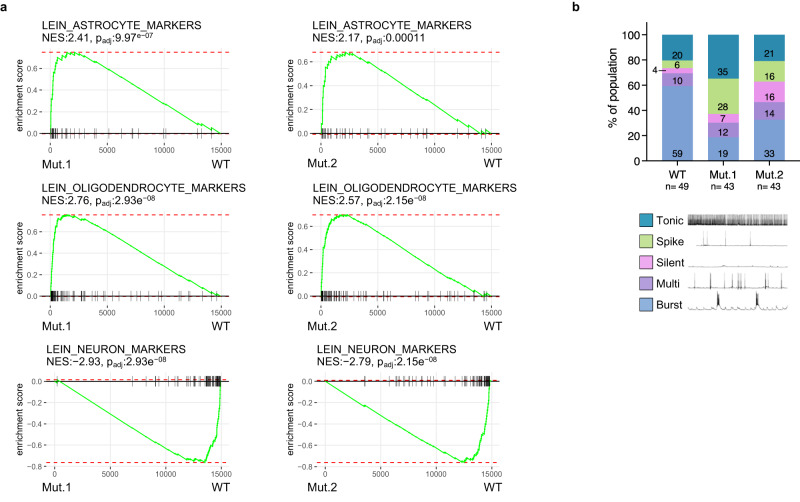
Mutant TOP1 mESCs showed altered differentiation. **a** GSEA enrichment plots from RNA-seq on neuronal cultures derived from Mut.1(left column) or Mut.2 (right column), compared with WT. NES, normalized enrichment score. *p*-values were calculated from gene set enrichment analysis (see details in ‘Methods’). **b** Distribution of mutant and WT neurons according to firing patterns from whole cell patch clamp experiments. Numbers in bars are percentages of each population. *n* = 49 (WT), *n* = 43 (Mut.1), *n* = 43 (Mut.2) cells patched. Source data are provided as a Source data file.

Defects in global transcription negatively impacts early embryonic development^6^. Therefore, we wanted to know whether the hypertranscription phenotype in mutant mESCs would impact its differentiation. In light of TOP1’s known roles in neuronal gene regulation^24,45^, we differentiated mutant and wild-type mESCs into neurons for comparison^46^. By RNA-seq and GSEA, we found oligodendrocyte and astrocyte markers to be upregulated while neuronal markers to be downregulated in mutants relative to wild-type (Fig. 6a). This suggests that differentiation is affected in the hyper-transcribing mutant mESCs. To substantiate this, we characterized the neurons by patch clamp where we identified five distinguishable neuronal subtypes based on firing patterns. For wild-type, bursting neurons are the dominant type (Fig. 6b). In comparison, both mutants clearly have reduced proportions of bursting neurons and increased proportions of spiking neurons. We determined that the distribution of neuronal subtypes between wildtype and mutant cells are significantly different (chi-square test for significance; chi-square = 17.09, df = 4, *p* value = 0.0019). This again indicates that differentiation of mutant mESCs is affected. Therefore, increased transcription in mESCs, as a result from a mutation at the secondary DNA binding site of TOP1, has functional consequences on cellular physiology and function.

## Discussion

Our study revealed TOP1 to be an important player in maintaining global transcription levels. This is dependent on a highly conserved DNA binding surface that is distal to its catalytic site, which is required for downregulating transcription. We identified a key DNA-interacting residue R546 on this surface of human TOP1 (R548 in mouse TOP1), which when mutated not only abrogated the ability of TOP1 to prevent hypertranscription in mESCs, but also reduced TOP1 chromatin occupancy. Interestingly, genomic supercoiling is also affected in the mutant cells. We verified that the mutation does not affect catalytic activity of TOP1 but negatively impacts its ability to bind and cause DNA conformational changes in vitro. The hyper-transcribing mutant mESCs are impacted in their differentiation to neurons, which underscores the importance of keeping transcription output at appropriate levels in cellular physiology.

### A role of TOP1 in transcription repression

The importance of global transcriptional change in development and disease is being increasingly recognized, especially following the advent of methods that enabled their detection in primary human tumour samples and adult tissues^2–4^. As aforementioned, little is known about how absolute transcription levels are regulated, and most known regulators to date are those that initiate hypertranscription^7,9,47,48^. In this regard, our finding that TOP1 exerts a gene repressive function to counteract transcription overdrive is significant.

Interestingly, two recent studies have highlighted the occurrence of widespread hypertranscription in aggressive human cancers^3,4^. Unexpectedly, one of them revealed that the hypertranscriptional phenotype is likely more commonly driven by loss of transcriptional suppression, than by gain in transcriptional activation^3^. Building upon our findings that TOP1 plays a role in transcriptional suppression, we sought to determine whether there is correlation between *TOP1* expression levels and RNA content in tumours^4^. Notably, in fourteen of the TCGA cancer types that were amenable to our analysis, four exhibited a negative correlation between TOP1 expression levels and RNA content, while only one showed a positive correlation (Supplementary Fig. 8). These results suggest that TOP1 overexpression may indeed play a role in transcriptional suppression in tumours. It is worth noting that previous studies in yeast have reported TOP1’s involvement in the transcriptional repression of telomere-proximal genes during exponential growth phase, and the induction of global transcriptional repression during stationary phase^22,23^. Therefore, TOP1-dependent transcriptional downregulation may have implications in diverse biological contexts.

### A non-catalytic function of TOP1 in transcription regulation

We have noted in the beginning that TOP1 plays various roles in the transcription cycle^17^. Most of these are generally attributed to its enzymatic function. However, few studies both in vivo and in vitro have alluded to TOP1 having additional activities that are not dependent on it catalytic activity^19,24–26^.

Prior to our studies, the secondary DNA binding site of TOP1 was only characterized in vitro where DNA binding is linked to the induction and recognition of supercoiled DNA^30,31^. Our study expands on this previous knowledge by investigating the secondary DNA binding site in vivo, specifically its role in transcription. Our findings indicate that TOP1 through binding at juxtaposed DNA segments could form a molecular connection between supercoiling level detection and transcriptional regulation. In our study, we found decreased negative genomic supercoiling in TOP1 mutant mESCs (Fig. 3e) which likely arises in part from the combined result of elevated transcription and reduced mutant TOP1 binding on chromatin. It is important to note that genomic supercoiling in vivo is highly dynamic and is influenced by multiple simultaneous activities on the DNA. Of note, hypertranscription has been linked to protein factors whose activities would have direct consequences on genomic supercoiling or chromatin structure, such as nucleosomes in ageing yeast and the chromatin remodeler Chd1 in mESCs^13,49^. Investigating the interplay of the various factors that were previously implicated in hypertranscription could provide a more holistic understanding on how global transcription levels could be governed through DNA and chromatin structures.

We recognize that the R548Q mutation in mESCs could have other functional consequences that contribute to the increased transcription phenotype observed in mESCs. While we cannot dismiss the possibility of other effects arising directly or indirectly from the mutation, our comprehensive approach, which includes genetic knock-in experiments and in vitro studies with purified TOP1 protein, provides strong evidence for the impact of the mutation on DNA binding and transcription. On a similar note, while our study focuses on RNAP2-dependent transcription and mRNA levels, it is plausible that the TOP1 mutation also affects transcription by RNA Polymerases I and III, given TOP1’s known roles in transcription by these polymerases.

### Opposing but coordinated regulatory influences of TOP1 on transcription

While our study highlights TOP1 as a transcriptional repressor, TOP1 is also an important transcriptional activator as demonstrated in many previous studies (for example see refs. ^20,35^). Our findings further reveal that the opposing influences of TOP1 on transcription are distinct and can be uncoupled, as the loss of transcriptional restraint due to R548Q mutation did not affect TOP1’s ability in enabling transcription (Fig. 3b and Supplementary Fig. 5b). This suggests that TOP1 can coordinate these activities in a manner that ensures proper regulation of transcription levels, preventing them from exceeding or falling below appropriate thresholds. In essence, TOP1 acts as a safeguard that maintains total transcription within the appropriate range in cells.

**Fig. 7:**
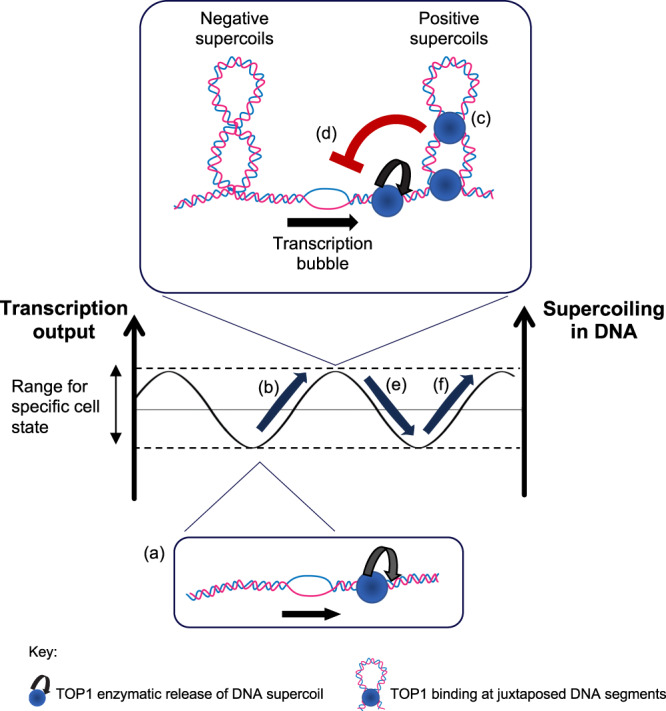
Model for how TOP1 safeguards transcription output levels. As DNA helix is being unwound during transcription (**a**), DNA helical stress concomitantly increases with transcription (**b**) and topological structures such as plectonemes are being formed ahead and behind the transcription bubble, if torsional stress is not sufficiently relieved by catalytic activity of TOP1. Additional TOP1 proteins, through simultaneous DNA binding at both its DNA binding sites, could preferentially bind at juxtaposed DNA helices on the plectonemes and form compact DNA structures that could further torsionally constrain the DNA (**c**). This built up of superhelical stress in DNA leads to deceleration in transcription (**d**), thereby preventing further increase in supercoiling. The decrease in transcription subsequently reduces the amount of helical stress being generated (**e**) and the eventual lifting of the restraint placed on transcription. This then allows transcription to increase again (**f**), thereby preventing transcription from falling below appropriate levels. The cycle then repeats.

Putting all our observations together, we propose a model for how TOP1 could safeguard transcriptional output in cells (Fig. 7). As DNA helix is being unwound during transcription (Fig. 7a), DNA helical stress concomitantly increases with transcription (Fig. 7b) and topological structures such as plectonemes are being formed ahead and behind the transcription bubble, if torsional stress is not sufficiently relieved by catalytic activity of TOP1. Additional TOP1 proteins, through simultaneous DNA binding at both its DNA binding sites, could preferentially bind at juxtaposed DNA helices on the plectonemes and form compact DNA structures that could further torsionally constrain the DNA (Fig. 7c). This built up of superhelical stress in DNA leads to deceleration in transcription (Fig. 7d), thereby preventing further increase in supercoiling. The decrease in transcription subsequently reduces the amount of helical stress being generated (Fig. 7e) and the eventual lifting of the restraint placed on transcription. This then allows transcription to increase again (Fig. 7f), thereby preventing transcription from falling below appropriate levels. The cycle then repeats. Therefore, this together creates a thermostat-like monitoring system that regulates absolute transcription levels through sensing and modulating changes in DNA supercoiling levels.

In conclusion, we have discovered a transcriptional regulatory role of TOP1 that is achieved through DNA binding at its secondary DNA binding site. Our findings not only ascribed in vivo relevance for the secondary DNA binding site of TOP1, but also added a new dimension to the current understanding of TOP1 as a multi-faceted regulator of transcription.

## Methods

### Molecular dynamics simulations

The crystal structure of human TOP1(Y723F) bound to DNA (PDB code 1A36)^50^ was used as the starting structure for molecular dynamics (MD) simulations. Missing loop residues in TOP1 were added using the ModLoop web server^51^. To generate the structure of the TOP1–DNA cleavage complex (TOP1cc), the Y723F mutation was reversed, the P-O5’ bond in the phosphate group connecting nucleotides T10 and A11 on one strand of the DNA duplex was cut, and the phosphate group was rotated towards Y723 using PyMOL (v.1.3)^52^. The LEaP module of AMBER18^53^ was used to form the bond between the phosphorus atom and hydroxyl oxygen of Y723. The N-terminus of TOP1 was capped by an acetyl group. To model the structure of TOP1cc bound to a linear DNA fragment at the surface secondary DNA binding site, the coordinates of the secondary DNA were extracted from the crystal structure of *Deinococcus radiodurans* TOP1B in complex with a duplex DNA (PDB code 3M4A)^30^ onto the PDB structure 1A36 after structural alignment of their secondary DNA binding motifs. A total of three systems were set up: wild-type (WT) TOP1cc bound to secondary DNA, K466A-K468A-K545A-K549A mutant TOP1cc bound to secondary DNA, and R546Q mutant TOP1cc bound to secondary DNA. The mutant systems were set up using the final trajectory structure from a stable MD simulation run of WT TOP1cc bound to secondary DNA. This structure was also used to initiate four independent replicate MD simulation runs with different initial atomic velocities. The R546Q mutation was introduced using the ModLoop web server. PDB2PQR(v.1.7)^54^ was used to determine the protonation states of residues. Each system was then solvated with TIP3P water molecules^55^ in a periodic truncated octahedron box such that its walls were at least 10 Å away from the solute, followed by charge neutralization with sodium ions.

Using different initial atomic velocities and seeds for the pseudorandom number generator, four independent explicit-solvent MD simulations were carried out on each of the TOP1–DNA systems. Energy minimizations and MD simulations were performed with the PMEMD module of AMBER18 using the ff14SB force field^56^ for the protein and the OL15^57^ force field for DNA. Atomic charges for the covalently bound Y723 and thymine nucleotide were derived using the R.E.D. Server^58^ by fitting restrained electrostatic potential (RESP) charges^59^ to a molecular electrostatic potential computed by the Gaussian 16 program^60^ at the HF/6-31 G* level of theory. All bonds involving hydrogen atoms were constrained by the SHAKE algorithm^61^, allowing for a time step of 2 fs. A cut-off distance of 9 Å was implemented for nonbonded interactions. The particle mesh Ewald method^62^ was used to treat long-range electrostatic interactions under periodic boundary conditions. The protein and DNA non-hydrogen atoms were kept fixed with a harmonic positional restraint of 2.0 kcal/mol/Å^2^ during the minimization and equilibration steps. Energy minimization was carried out using the steepest descent algorithm for 1000 steps, followed by another 1000 steps with the conjugate gradient algorithm. The systems were then heated gradually to 300 K over 50 ps at constant volume before equilibration at a constant pressure of 1 atm for another 50 ps. Subsequent unrestrained equilibration (2 ns) and production (200 ns) runs were carried out at 300 K using a Langevin thermostat^63^ with a collision frequency of 2 ps^−1^, and 1 atm using a Berendsen barostat^64^ with a pressure relaxation time of 2 ps. Because of the low sequence similarity between human TOP1 and *Deinococcus radiodurans* TOP1B at the secondary DNA binding interface, there were several atomic clashes between the protein and DNA in the initial model. As a result, the secondary DNA remained stably bound to WT TOP1 at the end of only one replicate run. Using the final trajectory structure of this stable run as the initial structure, four independent replicate MD simulation runs with reassigned atomic velocities were performed for 200 ns.

Binding free energies for the TOP1cc–secondary DNA complexes were calculated using the molecular mechanics/generalized Born surface area (MM/GBSA) method implemented in AMBER18. Two hundred equally spaced snapshot structures were extracted from the last 60 (K466A-K468A-K545A-K549A mutant), 80 (WT) and 100 ns (R546Q mutant) of the trajectories, during which the secondary DNA remained stably bound to TOP1, and their molecular mechanical energies calculated with the sander module. The polar contribution to the solvation free energy was calculated using the modified Generalized Born (GB) model described by Onufriev et al.^65^, with the solute dielectric constant set to 4 and the exterior dielectric constant set to 80. The nonpolar contribution was estimated from the solvent accessible surface area using the molsurf program with *γ* = 0.0072 kcal Å^−2^ and *β* = 0. The entropy contribution was ignored as it has been shown to be unnecessary for ranking the binding affinities of structurally similar ligands.

For hydrogen bonds between TOP1 and secondary DNA shown in Table 1, only those that are present for more than 5% of the last 80 ns of the replicate run with the lowest binding free energy are shown, and only hydrogen bonds for which the angle between the donor and acceptor heavy atoms is greater than or equal to 135° and their distance is less than or equal to 3.5 Å are counted.

VMD (v.1.9.2)^66^ was used for visualization of the MD simulations.

### Protein sequence alignment

Protein sequences for TOP1 from human, mouse, *Xenopus laevis*, *Caenorhabditis elegans*, *Saccharomyces cerevisiae*, *Drosophila melanogaster* and *Vaccinia* virus (strain Western Reserve, were obtained from Uniprot^67^ and aligned using PRALINE multiple sequence alignment webtool (https://www.ibi.vu.nl/programs/pralinewww/)^68^.

### Cell culture

293T cells were cultured in DMEM (Hyclone Cat #:SH30022.0) containing 15% fetal bovine serum (Hyclone Cat #:SV30160.03), GlutaMax (Life Tech Cat #:35050061) and non-essential amino acids.

mESCs were cultured in KnockOut DMEM (Life Tech Cat #:10829018) containing 15% ES-qualified fetal bovine serum (Life Tech Cat #:16141079), L-glutamine, non-essential amino acids, penicillin/streptomycin, 2-mercaptoethanol, LIF, 1 µM PD0325901 and 3 µM CHIR99021 (2i) on 0.1% gelatine-coated tissue culture dishes.

### Generation of overexpression constructs for human TOP1 variants

Wild-type human *TOP1* coding sequence (CDS) was amplified from cDNA library of MCF7 cells and sequence-verified by Sanger sequencing. *TOP1* CDS was cloned into a vector previously modified to create N’−3xHA-tagged fusion proteins. To generate TOP1(4K-A), TOP1(R546Q) and TOP1(Y723F) variant coding sequences, we performed site-directed mutagenesis using the primers indicated in using the Gibson Assembly cloning method. The constructs were verified by Sanger sequencing. The correct coding sequences were transferred into pLVX vector (Takara), previously modified include IRES EGFP sequence as a reporter for overexpression, to result in pLVX-N’3xHA-*TOP1* (WT, 4K-A, R546Q, or Y723F variant)-IRES-GFP. To generate overexpression constructs without the GFP reporter for EU labelling experiments, the coding sequences were transferred to pCI overexpression vector (Promega Cat #: E1841). Sanger sequencing results were viewed and aligned on Benchling webtool (https://www.benchling.com).

### Overexpression of TOP1 variants in 293T cells

For RNA-seq and RNA quantification experiments, 293T cells were transfected with pLVX-N’3xHA-*TOP1* (WT, 4K-A, R546Q or Y723F variant)-IRES-GFP constructs or empty vector using FuGENE HD (Promega Cat #: E2312) according to the manufacturer’s protocol. At 70 h post transfection, cells were harvested and sorted for GFP positive cells on a Mo-Flo running on Summit (v.5.4) software. The gating strategy is described in Supplementary Fig. 9.

For EU labelling, 293T cells were transfected with pCI-TOP1 **(**WT, or R546Q variant) constructs or empty vector using FuGENE HD. Cells were processed at 48 h post transfection.

### RNA isolation and RNA-seq library preparation

For 293T cells, 1.0×10^5^ GFP-positive cells were sorted directly into RLT buffer supplemented with 2-mercaptoethanol and processed using RNeasy Mini Kit (Qiagen Cat #: 74104) for RNA isolation. RNA concentrations were determined using Qubit RNA Broad Range assay kit (Thermofisher Cat #: Q10210).

For mESCs, cells were counted manually on the haematocytometer or using Countess II automated cell counter (ThermoFisher). Equal number of cells across samples were used in each RNA isolation experiment. Cell pellets were dissolved in TriReagent and isolated using Direct-Zol RNA miniprep kit (Zymo Cat #: R2053). Equal volumes of RNA preparations (which originated from equal number of cells) were each mixed with 1 μl of 1:100 diluted Mix 1 of ERCC RNA Spike-In (Life Technologies Cat. # 4456739). The mixture is subjected to ribosomal RNA depletion using NEBNext® rRNA Depletion Kit (Human/Mouse/Rat) (NEB Cat # E6310) and library construction using NEBNext® Ultra™ II Directional RNA Library Prep Kit for Illumina (NEB Cat # E7760) according to manufacturer’s protocol.

For neuronal samples, cells were collected following detachment using Accutase (Stemcell Cat #: 7920). TriReagent was added to collected cells and RNA was isolated using Direct-Zol RNA miniprep kit. 500 ng of RNA was used for RNAseq library construction as described above, starting from the rRNA depletion step.

Pooled libraries were sequenced using the Illumina HiSeq 2000 System.

### Western blot

Cells were lysed in urea lysis buffer (50 mM Tris-HCl pH7.9, 10 mg/mL CHAPS, 480 mg urea) supplemented with protease inhibitor cocktail (Aprotinin/Leupeptin/Pepstatin (A/L/P), PMSF, and sodium butyrate), pre-cleared by centrifugation and subjected to standard Western blotting procedures. The following primary and secondary antibodies were used: anti-TOP1 (Bethyl, Cat #: A302-590A, 1:1000 or 1:2000), anti-RNA polymerase II CTD repeat YSPTSPS (PhosphoS2) (Abcam, Cat #: ab5095, 1:1000), anti-HA (HA.11) (Biolegend, Cat #: 901501, 1:4000), anti-actin (C-4) (Santa-Cruz, sc-47778, 1:1000 or 1:2000), anti-AFF4 (Proteintech 14662-1-AP, 1:20,000), anti-gamma H2A.X (phospho S139) antibody (Abcam, ab2893, 1 μg/mL), goat anti-Rabbit IgG HRP-Conjugated (Bethyl, Cat #: A120-201P, 1:20,000), goat anti-Mouse IgG HRP-Conjugated (Bethyl, Cat #: A90-516P, 1:20,000). The antibodies are also summarized in Supplementary Data 3.

### Immunofluorescence imaging

293T cells were transfected with pLVX-overexpression or vector control and seeded onto imaging chambers (Ibidi Cat #: 81156) pre-coated with poly-D-lysine. At about 70 h post-transfection, cells were fixed with 3.7% formaldehyde in DPBS for 15 min at room temperature, washed twice with DPBS, permeabilized and blocked in DPBS supplemented with 0.5% Triton X and 1% BSA for 15 min at room temperature, and incubated overnight at 4 degC with primary antibodies anti-gamma H2A.X (phospho S139) (Abcam, Cat #: ab2893, 1:1000), and anti-GFP (Abcam, Cat #: 6673, 1:500). On the following day, the imaging chambers are washed thrice with DPBS, and incubated with secondary antibodies donkey anti-Goat IgG (H + L) Alexa Fluor® 488 conjugate (Thermofisher, Cat #: A11055, 1:500) and donkey anti-Rabbit IgG (H + L) Alexa Fluor® 594 conjugate (Thermofisher, Cat #: A21207, 1:250), and DAPI (1 μg/mL) for 1.5 h at room temperature. The chambers were washed thrice with DPBS before imaging using Zeiss LSM700 confocal microscope.

### Cell cycle analysis

293T cells transfected with either pLVX-N’3xHA-*TOP1* (WT)-IRES-GFP or empty vector were harvested 70 hours post-transfection and stained with Hoescht 33342 (20 μg/mL) in PBS supplemented with 5% FBS for 20 min at 37degC. Flow cytometry was performed on a BD Symphony A5 analyser running on FACSDiva (v.9.8). Cells were first gated for GFP positive signal, which marks transfected cells, before being analysed for Hoescht 33342 staining. The gating strategy is described in Supplementary Fig. 10. Cell cycle analysis was performed on FlowJo (v.10.8.2).

### 5-ethynyl uridine (EU) labelling of nascent transcripts

The assay was performed using Click-iT RNA imaging kit (Invitrogen Cat #: 10329) according to manufacturer’s protocol. Briefly, 293T cells were transfected with pCI-overexpression or vector control construct and seeded onto imaging chambers (Ibidi Cat #: 81156) pre-coated with poly-D-lysine. Forty-eight hours later, cells were fed with media supplemented with 1 mM EU for 1 h, then fixed and labelled by Click-iT as per manufacture’s protocol. Cells were then stained with DAPI for immunofluorescence imaging. Images were taken on Zeiss LSM700 confocal microscope. Three regions were randomly selected for each condition in each experiment. Nuclear segmentation using DAPI signal and determination of mean EU signal intensity were carried out using the surface function in Imaris software (Oxford Instruments).

### Generation of homozygous Top1(R548Q) mESC lines

E14 mESCs were transfected with CRISPR–Cas9 single-guide RNA cloning vector pX458 (Addgene plasmid # 48138) containing single-guide RNA against *Top1* and a single-stranded oligo DNA donor (see Supplementary Data 2 for sequences, and for sequences of primers described here). Transfected cells were sorted for GFP signal and expanded. Single colonies were picked for genotyping via Sanger sequencing as follow: the genomic region of interest on *Top1* was amplified using the primers gDet9 and gDet10, and the product was sequenced using the primer gDet11.

### Precision nuclear run-on sequencing (PRO-seq)

Ten million mESCs were harvested, washed 1× in cold PBS, and resuspended into single-cell suspension in 250 μl ice cold buffer W (10 mM Tris -HCl (pH 8.0), 10 mM KCl, 250 mM sucrose, 5 mM MgCl_2_, 1 mM EGTA, 0.5 mM DTT, 10% (v/v) glycerol, supplemented with 1× protease inhibitor cocktail (Merck, Cat # 11873580001) and SUPERase-IN RNase inhibitor at 10 μL/50 mL buffer (Thermofisher, Cat # AM2696). 5 mL of ice-cold buffer P (10 mM Tris-HCl (pH8.0), 10 mM KCl, 250 mM sucrose, 5 mM MgCl_2_, 1 mM EGTA, 0.1% (v/v) NP40, 0.5 mM DTT, 0.05% (v/v) Tween-20, 10% (v/v) glycerol, supplemented with protease inhibitor cocktail and SUPERase-IN RNase inhibitor) was added to cells and the suspension was incubated on ice for 5 min for permeabilization. The cells were collected by centrifugation at 400 × *g*, for 4 min at 4 degC, washed with 5 mL of ice-cold buffer W, and resuspended in 500 μL buffer F (50 mM Tris-HCl (pH8.0), 40% (v/v) glycerol, 5 mM MgCl_2_, 1.1 mM EDTA, 0.5 mM DTT, supplemented with SUPERase-IN RNase inhibitor). The cells were checked under the microscope for the extent of permeabilization before snap-freezing.

PRO-Seq library construction and data analysis was performed by the Nascent Transcriptomics Core at Harvard Medical School, Boston, MA, as follows.

### PRO-seq library construction

Aliquots of frozen (−80 °C) permeabilized cells were thawed on ice and pipetted gently to fully resuspend. Aliquots were removed and permeabilized cells were counted using a Luna II, Logos Biosystems instrument. For each sample, 1 million permeabilized cells were used for nuclear run-on, with 50,000 permeabilized Drosophila S2 cells added to each sample for normalization. Nuclear run on assays and library preparation were performed essentially as described in Reimer et al.^69^. with modifications noted: 2× nuclear run-on buffer consisted of (10 mM Tris (pH 8.0), 10 mM MgCl_2_, 1 mM DTT, 300 mM KCl, 20 uM/each biotin-11-NTPs (Perkin Elmer), 0.8 U/μL SuperaseIN (Thermo), 1% sarkosyl). Run-on reactions were performed at 37 °C. Random hexamer extensions (UMIs) were added to the 3’ end of the 5’ adapter and 5’ end of the 3’ adapter. Adenylated 3’ adapter was prepared using the 5’ DNA adenylation kit (NEB) and ligated using T4 RNA ligase 2, truncated KQ (NEB, per manufacturers’ instructions with 15% PEG-8000 final) and incubated at 16 °C overnight. 180 μL of betaine buffer (1.42 g of betaine brought to 10 mL) was mixed with ligations and incubated 5 min at 65 °C and 2 min on ice prior to addition of streptavidin beads. After T4 polynucleotide kinase (NEB) treatment, beads were washed once each with high salt, low salt, and 0.25× T4 RNA ligase buffer (NEB) and resuspended in 5’ adapter mix (10 pmol 5’ adapter, 30 pmol blocking oligo, water). 5’ adapter ligation was per Reimer but with 15% PEG-8000 final. Eluted cDNA was amplified 5-cycles (NEBNext Ultra II Q5 master mix (NEB) with Illumina TruSeq PCR primers RP-1 and RPI-X) following the manufacturer’s suggested cycling protocol for library construction. A portion of PCR was serially diluted and for test amplification to determine optimal amplification of final libraries. Pooled libraries were sequenced using the Illumina NovaSeq platform.

### PRO-seq data analysis

Dual, 6nt Unique Molecular Identifiers (UMIs) were extracted from read pairs using UMI-tools (v 1.1.4; DOI:10.1101/gr.209601.116). Read pairs were trimmed using cutadapt (v1.14; 10.14806/ej.17.1.200) to remove adapter sequences (-O 1 --match-read-wildcards -m {20,26}). The UMI length was trimmed off the end of both reads to prevent read-through into the mate’s UMI, which will happen for shorter fragments. An additional nucleotide was removed from the end of read 1 (R1), using seqtk trimfq (v1.3-r119-dirty; https://github.com/lh3/seqtk), to preserve a single mate orientation during alignment. The paired end reads were then mapped to a combined genome index, including both the spike (dm6) and primary (mm10) genomes, using bowtie2 (v.2.2.9). Properly paired reads were retained. These read pairs were then separated based on the genome (i.e. spike-in vs primary) to which they mapped, and both these spike and primary reads were independently deduplicated, again using UMI-tools. Reads mapping to the reference genome were separated according to whether they were R1 or R2, sorted by name via samtools (v1.4), and subsequently converted to bedGraph format using a custom script bowtie2stdBedGraph.pl (see Code Availability statement) that counts each read once at the exact 3’ end of the nascent RNA. Because R1 in PRO-seq reveals the position of the RNA 3’ end, the ‘+’ and ‘−’ strands were swapped to generate bedGraphs representing 3’ end positions at single nucleotide resolution. Combined bedGraphs were generated by summing counts per nucleotide across replicates for each condition.

### Refinement of gene annotation (proTSScall)

PRO-seq reads were used to identify active transcription start sites using a custom script, proTSScall (see Code Availability statement). Briefly, PRO-seq 3’ read bedGraphs for ‘+’ and ‘−’ strands were separately combined across samples and the composite read counts were assigned to TSS-proximal windows (TSS to +150nt) using the same filtered TSS annotation described above. TSSs with ≤9 counts in this window are deemed ‘inactive’ and the remaining TSSs, deemed ‘active’, are collapsed to yield 1 dominant TSS per gene, defined as the one with the highest TSS-proximal read count—if the highest read count is shared among multiple transcripts, the TSS furthest upstream, in a strand-aware fashion, is called dominant. Dominant TSSs sharing the same start position are deduplicated as follows: (1) if start positions are equal, the TSS with the longest associated annotated transcript is called dominant, (2) if start positions and transcript lengths are both equal, the TSS associated with the lowest Ensembl gene ID (numerical portion) is dominant.

### Differential expression analysis

Reads were summed within the TSS to TES window for each active gene using the using the make_heatmap script (see ‘Code availability’ statement), which counts each read one time, at the exact 3’ end location of the nascent RNA. DEseq2, was used to determine statistically significant differentially expressed genes (DEGs), based on the sample size factors obtained from spike-in reads for normalization, and Wald test with correction for multiple testing using the Benjamini and Hochberg FDR method.

### Chromatin immunoprecipitation (ChIP) and ChIP-seq library preparation

For TOP1-ChIP, mESCs were harvested and double-crosslinked in suspension with disuccinimidyl glutarate (DSG) (Life Technologies) and formaldehyde (FA) (Sigma) according to Tian et al.^70^. Briefly, cells were incubated with 2 mM DSG in PBS for 45 min at room temperature, washed with PBS thrice, and incubated with 1% FA in PBS for 10 minutes at room temperature. FA crosslinking was quenched by addition of glycine to final concentration of 500 mM and incubation at room temperature for 5 min. Cells were washed in PBS and snap frozen for storage at −80 degC until ChIP was performed.

For RNAP2(S2P) ChIP, mESCs were subjected to crosslinking in 1% FA for 10 min at room temperature.

To perform ChIP, crosslinked cells were incubated in cell lysis buffer (5 mM Tris-HCl (pH 8.0), 85 mM KCl, 0.5% NP-40), then in nuclear lysis buffer (50 mM Tris-HCl (pH 8.0), 10 mM EDTA pH 8.0, 1% SDS, 0.75% Triton-X). Nuclear extract was diluted with an equal volume of IP buffer (16.7 mM Tris-HCl (pH 8.0), 1.2 mM EDTA pH 8.0, 167 mM NaCl, 0.01% SDS, 1.1% Triton-X, 0.1% Tween-20) and sonicated in Bioruptor (Diagenode) to shear chromatin. Chromatin extract was pre-cleared by centrifugation, followed by nutation with Protein G Dynabeads (Life Technologies) for TOP1 ChIP, or Protein A Dynabeads (Life Technologies) for RNAP2(S2P) ChIP for 2 h at 4 degC. Chromatin extract equivalent to 10 or 30 million cells were used for immunoprecipitation with anti-TOP1 antibody (Bethyl, Cat #: A302-590A, 7.5 μg/IP) -conjugated to Protein G Dynambeads, or anti-RNAP2(S2P) antibody (Abcam, Cat #:ab5095, 4 μg/IP)- conjugated to Protein A Dynabeads, overnight. Beads were washed in low salt buffer (20 mM Tris-HCl (pH 8.0), 2 mM EDTA pH 8.0, 150 mM NaCl, 0.1% SDS, 1% Triton-X, 0.05% Tween-20), high salt buffer (20 mM Tris-HCl (pH 8.0), 2 mM EDTA pH 8.0, 500 mM NaCl, 0.1% SDS, 1% Triton-X, 0.05% Tween-20), LiCl wash buffer (10 mM Tris-HCl (pH 8.0), 1 mM EDTA pH 8.0, 250 mM LiCl, 1% NP40, 0.1% sodium deoxycholate, 0.05% Tween-20), and TE buffer. Chromatin complex was eluted in elution buffer (50 mM NaHCO_3_, 1% SDS), treated with RNaseA and proteinase K, and reversed crosslinked. DNA was purified using SPRI method. All buffers except for TE and elution buffers were supplemented with protease inhibitor cocktail (A/L/P, PMSF, and sodium butyrate) before use.

Immunoprecipitated DNA and DNA from relevant input materials were made into ChIP-seq libraries using NEBNext Ultra II DNA kit (NEB Cat #: E7645) according to manufacturer’s protocol. Pooled libraries were sequenced using the Illumina HiSeq 2000 System.

### Co-immunoprecipitation (co-IP) experiment

Nuclear extracts from mESCs were prepared as follows: cells were collected and resuspended in ice-cold TMSD buffer (20 mM HEPES (pH 7.5), 5 mM MgCl_2_, 250 mM sucrose, 1 mM DTT) for swelling. Then, cells were resuspended and incubated with ice-cold TMSD buffer supplemented with 0.1% NP-40 to release the nuclei. The nuclei were collected by centrifugation and lysed through incubation in ice-cold low salt lysis buffer (20 mM Tris-Cl pH 7.9, 420 mM KCl, 1.5 mM MgCl2, and 0.2 mM EDTA) at 4 degC with constant rotation, followed by a three-cycle sonication in a Bioruptor (Diagenode). Thereafter, the nuclear lysate was pre-cleared by centrifugation, and the supernatant (‘420 mM fraction’) was transferred into a fresh microfuge tube. The insoluble pellet was further extracted with ice-cold high salt lysis buffer (20 mM Tris-Cl (pH 7.9), 700 mM KCl, 1.5 mM MgCl_2_, and 0.2 mM EDTA), pre-cleared by centrifugation, and the supernatant collected (‘700 mM fraction’). Both ‘420 mM fraction’ and ‘700 mM fraction’ were dialysed in BC100 (50 mM Tris-Cl (pH 7.9), 2 mM EDTA, 10% glycerol, 100 mM KCl, and 0.2 mM PMSF), and combined. 1 mg of this extract was additionally pre-cleared through incubation with 30 μl Protein A-Dynabeads before used for each IP. All buffers except for BC100 for dialysis were supplemented with protease inhibitor cocktail (A/L/P, PMSF, and sodium butyrate).

For each IP, 4 μg of TOP1 antibody (Bethyl, Cat #: A302-590A) pre-coupled to 30 μl Protein A Dynabeads was used. Immunoprecipitation was carried out overnight with constant rotation at 4 degC. This was followed by four washes in BC200 (50 mM Tris-Cl (pH 7.9), 2 mM EDTA, 10% glycerol, 200 mM KCl, 0.1% NP-40, 0.05% Tween-20, supplemented with protease inhibitors). Immunoprecipitations were finally eluted with NuPAGE™ LDS Sample Buffer (Invitrogen, Cat #: NP0007). Elutions and matching input nuclear extracts were then subjected to Western blot analyses.

### Biotinylated-trimethylpsoralen (bTMP) supercoiling assay

The assay was performed as previously described^35,36^. Briefly, mESCs were seeded into imaging chambers pre-coated with 0.1% gelatin. Twenty-four hours later, cells were fed with media supplemented with 1 μM aphidicolin (Sigma-Aldrich, Cat#: A0781) for two hours; aphidicolin inhibits DNA polymerases, thereby prevents supercoiling that arise from DNA replication.

Cells were then washed with DPBS, permeabilized with 0.1% Tween-20 in DPBS for 15 minutes, and incubated with 0.3 mg/mL EZ-Link Psoarlen-PEG3-Biotin (Thermo, Cat #:29986) for 15 min at room temperature. Cells were then exposed to 365 nM light (Vilber Lourmate UV lamp with 15 W bulbs) for 15 minutes for crosslinking. Cells were then washed twice with DPBS, fixed with cold 70% ethanol for 30 minutes at 4 degC, and washed twice with DPBS, before incubation with antibody Alexa Fluor 594 Streptavidin (Thermofisher, Cat #:S32356, 1:250) for one hour at room temperature in the dark. Cells were washed twice with DPBS, stained with DAPI for 10 min, and then imaged in a buffer containing 50% glycerol (Thermo, Cat #: 17904), 75 μg/mL glucose oxidase (Sigma Aldrich, cat #: G7141), 520 μg/mL catalase (Sigma Aldrich, Cat #: C3515), and 0.5 mg/mL Trolox (Sigma Aldrich, Cat #: 238813) on a FV3000 Confocal Laser Scanning Microscope running on FV31S-SW (v.2.4.1.198) or a Zeiss LSM 700 confocal microscope running on Zen Blue Software (v.3.3.89.0000). Three regions were randomly selected for each condition in each experiment. For each region, *z*-stack images were taken. Nuclear segmentation using DAPI signal and determination of mean psoralen staining intensity were carried out using the spots function in Imaris software.

### Plasmid relaxation assay

Purified TOP1(WT) and TOP1(R546Q) were custom ordered from Sino Biological. Briefly, histidine tagged TOP1 variants were expressed in insect cells infected with recombinant baculovirus and purified using Ni columns. The protein preps were supplied in buffer containing 20 mM Tris-HCl pH 7.5, 300 mM NaCl, 10% glycerol, 1 mM TCEP. Their purity and concentration were verified using Coomassie Blue staining and a BSA standard (Supplementary Fig. 11). The proteins were diluted in buffer containing 20 mM Tris-HCl pH7.5, 1 mM DTT, 1 mM EDTA, 10% glycerol just before use.

To determine salt optima of TOP1(WT) and TOP1(R546Q), 100 fmol TOP1 protein was incubated with 200 fmol supercoiled pBluescript KS(II)+ plasmid in 10 mM Tris HCl (pH 7.5), 1 mM EDTA, and indicated NaCl concentrations in 20 μl total volume for 2 min at 37 degC. Reactions were then quenched with 5× Stop buffer containing 5% Sarkosyl, 0.125% bromophenol blue, 25% glycerol, treated with proteinase K (1 mg/mL) at 37 degC for 1 h, loaded on 1% agarose/TBE gel, and run at 5–6 volts/cm for 2.5-3 hours. Gel was then stained with SybrGold (Invitrogen), destained and imaged on Gel Doc XR+ (v.5.2) (BioRad). For analyses, the bands in each lane were automatically detected and quantified as percentage of total bands in the same lane using ImageLab (v.6.1) (Biorad). Loss of supercoiled plasmid was used as an indicator of relaxation activity.

To determine rates of catalytic activities, the plasmid relaxation reaction contained 100 fmol TOP1 protein with either 400 fmol supercoiled pBluescript KS(II)+ plasmid (1 enzyme: 4 DNA), or 50 fmol supercoiled plasmid (2 enzyme: 1 DNA) in 10 mM Tris HCl (pH 7.5), 200 mM NaCl, 1 mM EDTA in 20 μL total volume. The reactions were incubated at 37 degC for a specified duration and quenched with 5× Stop buffer. Reactions were then treated with proteinase K and analysed by DNA gel electrophoresis as above.

### Magnetic tweezers experiments

To conduct single molecule stretching by magnetic tweezers, DNA molecules were labelled with biotin and digoxigenin at each end for surface attachment. Primers 5’- /5Biosg/CT AAT GCT GCT TGC TGT TCT −3’ and 5-‘ /5DigN/CCG CCC GCT TCT TTG AAT T −3’ (Integrated DNA Technologies) were mixed with the phage-λ DNA template and amplified using Q5 Hot Start polymerase (NEB). PCR products were purified using the PCR Purification Kit (ThermoFisher).

The flow channel was created using two #1 glass coverslips. The bottom coverslip was first functionalized with 3-Aminopropyltriethoxysilane (Sigma-Aldrich) and then treated with 1% glutaraldehyde (Sigma-Aldrich). Subsequently, anti-digoxigenin Fab fragments (ThermoFisher) were flushed through the channel. Finally, the channel was filled with a 1% BSA solution in 1× phosphate-buffered saline (PBS) buffer (pH 7.4) and incubated overnight at 4 degC to prevent nonspecific binding of DNA to the surface before being used in experiments.

Single molecule experiments were performed using a self-built magnetic tweezers setup, as described in Zhao et. al.^71^. In the experiments, a single DNA molecule was tethered between a streptavidin-coated paramagnetic bead with a diameter of 1 μm (Dynabeads MyOne) and a coverslip. Real-time tracking of bead height was achieved with a spatial resolution of approximately 2 nm and temporal resolution of 10 ms using CMOS camera (acA1300-200um—Basler ace) and LabVIEW 2015. To generate constant forces, a translational micromanipulator (L509, Physik Instruments) was employed to control the height of a pair of Neodymium magnets. To investigate DNA conformational change upon TOP1 binding, we performed force-decrease scans followed by force-increase scans at increasing TOP1 concentrations. At each force, the tethered bead was held for 20 s and the error bars are standard deviations of the bead height during the recording time window.

### Generation of cortical glutaminergic neurons from mESCs

mESCs were differentiated according to Bibel et. al.^46^ with modifications: For cellular aggregate (CA) generation, 3×10^5^ mESCs were plated in media containing equal parts of Advanced DMEM/F12 (Life Tech Cat #: 12634-028) and neurobasal medium (Life Tech Cat#: 21103-049), 15% Knock-out-serum-replacement (Life Tech Cat #: 10828-028), supplemented with L-glutamine, penicillin/streptomycin and 2-mercaptoethanol, in ultra-low culture dishes (Corning Cat #: C05/3262). Media was changed every day from day 2 onwards, and for days 4 to 8, 5 μM retinoic acid was added to media. On day 8, CAs were dissociated using Accutase (Stemcell Cat #: 7920) and strained through a 40 μm nylon cell strainer. Cells were plated in 1×10^5^/cm^2^ density in tissue culture dishes pre-coated with poly-D-lysine (ThermoFisher Cat #: A3890401) and laminin (~0.5 μg/cm^2^) (Thermofisher Cat #: 23017015) in N2 medium containing DMEM/F12 (Thermofisher Cat #: 1132003), N2 supplement (MerckMillipore Cat #: SCM012), 1 mM GlutaMAX (Thermofisher Cat #: 35050061) and 50 μg/mL BSA. N2 medium was changed 2 h after plating, and again after 24 h. After 48 h, media was changed to complete medium containing Neurobasal Plus supplemented with B27 Plus (Thermofisher Cat #: A3653401), 1.5 mM GlutaMAX, 5 ng/mL BDNF (Miltenyi Cat #: 130-093-811). Complete medium was changed every 4 days thereafter. Neurons were used for experiments on day 15 after CA dissociation.

### Patch clamp experiments

Neurons were recorded with internal solution (pipette solution) containing 130 mM K-gluconate, 10 mM KCl, 5 mM EGTA, 10 mM HEPES, 1 mM MgCl_2_, 0.5 mM Na_3_GTP, 4 mM Mg-ATP, 10 mM Na-phosphocreatine pH 7.4 (adjusted with KOH) and external solution containing: 10 mM glucose, 125 mM NaCl, 25 mM NaHCO_3_, 1.25 mM NaH_2_PO_4_.2H_2_O, 2.5 mM KCl, 1.8 mM CaCl_2_, 1 mM MgCl_2_, pH 7.4 (300–310 mOsm). Whole cell recording was performed on Clampex (v.10.7.0.3) on multiclamp700b amplifier (Molecular Device), low pass filtered at 1 kHz and the series resistance was typically <10 MΩ after >50% compensation. The P/4 protocol was used to subtract online the leak and capacitive transients. Analysis of neuronal firing profiles was performed using pCLAMP10.5 software suite (Molecular Devices).

### RNA-seq data analysis

Paired-end raw sequencing reads were trimmed with Trim Galore^72^ with parameters: *--trim-n –paired*. Cleaned reads were then mapped to either hg19 (293 T cells) or mm10 (mESC and mESC-derived neurons) reference genome, guided by the corresponding gene models obtained from Illumina iGenome website (https://sapac.support.illumina.com/sequencing/sequencing_software/igenome.html) using the RSEM pipeline (v1.1.11)^73^. For RNA-seq experiments with 293T cells and mESCs, differential gene expression analysis was carried out using DESeq2 (v1.16.1)^74^ with default settings except for the calculation of sample normalization factors, which was based on ERCC spike-in. For RNA-seq experiments for neurons, default parameters of DESeq2 were used. For experiments with 293T cells, differentially expressed genes (DEGs) are defined by fold change greater than two-fold difference in expression and *p* adjusted value ≤ 0.01 from Wald test, with correction for multiple testing using the Benjamini and Hochberg FDR method. For mESCs, DEGs are defined by fold change greater than 1.5 and *p* adjusted value ≤ 0.05. Volcano plots were generated using ggplot2 in R (v4.0.5)^75^.

### Gene set enrichment analysis

For RNA-seq from 293T cells, gene set enrichment analyses (GSEA) were carried out against the Molecular Signatures Database (MSigDB v7.4)^76,77^ using GSEA function in the package clusterProfiler^78^. For RNA-seq from mESC-derived neurons, mouse genes were first converted to corresponding human orthologs to take advantage of the diverse pathways collected in MSigDB. GSEA were then carried out on the converted human orthologs. *p* values were calculated based on one million permutations. For both analyses, pathways were considered significant if the *p*-adjusted value, following FDR-correction, was ≤0.05. NES enrichment plot was plotted using function plotEnrichment in the package fgsea^79^.

### ChIP-seq data analysis

Single- and paired-end raw sequencing reads were trimmed with Trim Galore with parameters: --trim-n (for single-end datasets) or --trim-n -–paired (for paired-end datasets). Cleaned reads were then mapped to mm10 obtained from Illumina iGenome website by Bowtie2 (v.2.2.9)^76^ with parameters: -N 1 -L 25 --no-mixed --no-discordant for paired-end and -N 1 -L 25 for single-end sequencing respectively. Only uniquely mapped reads with MAPQ > = 10 were kept and PCR duplicates were removed using SAMtools (v1.4)^77^. Biological replicate alignment files were merged.

It is important to ensure that differences in normalized ChIP-seq read counts accurately reflect absolute changes in binding in the context of global changes in binding. As such, we have followed the method of normalizing ChIP-seq experiments as reported by Wiegard et al.^80^. Briefly, reads that are mapped to intergenic regions for TOP1 and RNAP2(S2P) ChIP-seq can be considered non-specific background that can serve as an internal calibration across sequencing libraries. Thus, we determined the number of reads that fall into the intergenic regions to calculate a scaling factor for normalizing the datasets. To define intergenic regions, we first defined an extended gene region for every gene as including the 5 kb region before its transcription start site (TSS) and 5 kb region after its transcription termination site (TES). We then designated intergenic regions as regions that do not overlap with any extended gene region.

BigWig coverage were generated using ‘bamCoverage’ function from the package deepTools^81^ with parameters: -bl mm10-blacklist.bed -bs 20 -p 6 --scaleFactor sizefactor_calculated normalizeUsing None -b IP.bam --extendReads 200 for single-end sequencing, and -bl mm10-blacklist.bed -bs 20 -p 6 --scaleFactor sizefactor_calculated –normalizeUsing None -b IP.bam --extendReads for paired-end sequencing, respectively. Blacklist region ‘mm10-blacklist.bed’ was obtained from https://github.com/Boyle-Lab/Blacklist/blob/master/lists/mm10-blacklist.v2.bed.gz. BigWig files were visualized on Integrative Genomics Viewer (IGV 2.10.2)^82^. Metaplots were then generated by functions ‘computeMatrix’ and ‘plotProfile’ functions with default parameter setting.

TOP1 peaks were called using the ‘callpeak’ function of MACS2 as follow: for paired-end dataset, macs2 callpeak -t IP.rmdup.bam -c INPUT.rmdup.bam -f BAMPE -g mm --broad; for single-end dataset, macs2 callpeak -t IP.rmdup.bam -c INPUT.rmdup.bam -f BAM -g mm --broad --keep-dup all. ‘keep-dup all’ is used because the PCR duplicates have already been removed earlier in the pipeline. ChIPSeeker^83^ was used to determine overlap with genomic features.

### Gene expression ranking and classification

Expression values in FPKM were averaged across three biological replicates for WT mESC. Genes of no expression were defined as silent. For expressed genes, the 5th and 95th quantile FPKM values were calculated, and the gene expression was classified as High, Medium, and Low if the FPKM was above the 95th quantile, between 5th and 95th quantiles, and below the 5th quantile, respectively.

### Statistical analysis

No statistical methods were used to pre-determine the sample size. The statistical analyses for confocal imaging signals (Figs. 1g and 3e) were performed on Jupyter (v.6.5.4). The statistical analyses TOP1 plasmid relaxation activities (Fig. 4 and Supplementary Fig. 7), magnetic tweezer experiments (Fig. 5), Neuron firing patterns (Fig. 6b) and RNA content quantification (Supplementary Fig. 3) were performed on Prism (v. 9.5.0). The statistical tests used were indicated in the respective figure legends.

### Reporting summary

Further information on research design is available in the Nature Portfolio Reporting Summary linked to this article.

### Supplementary information

Supplementary Information

Description of Additional Supplementary Files

Supplementary Data 1

Supplementary Data 2

Supplementary Data 3

Reporting Summary

### Source data

Source Data

## Data Availability

The MD simulation input files and processed output trajectories have been deposited in Zenodo and are available at 10.5281/zenodo.8158854. The raw and processed sequencing data generated in this study have been deposited in the NCBI Gene Expression Omnibus (GEO) under accession number GSE207163. The crystal structures PDB code 1A36 and PDB code 3M4A were retrieved from Protein Data Bank [www.wwpdb.org]. Human hg19, mouse mm10 and fly dm6 reference genomes was obtained from GENCODE [https://www.gencodegenes.org/]. Molecular Signatures Database (MSigDB) was obtained from https://www.gsea-msigdb.org/gsea/index.jsp. All other data are available in the main article, Supplementary Information, and Source data. Source data are provided with this paper.

## Code availability

All custom scripts for PRO-seq data analysis are available at 10.5281/zenodo.5519915 (ref. ^84^), except for *proTSScall* that is available at 10.5281/zenodo.8298661 (ref. ^85^).
