# Supplementary material for: Transcriptional repression by a secondary DNA binding surface of DNA topoisomerase I safeguards against hypertranscription
Source: Nat Commun. 2023 Oct 13;14:6464. doi: 10.1038/s41467-023-42078-9 (PMC10576097; doi:10.1038/s41467-023-42078-9)
Supplement: Supplementary file 1 — Supplementary Information [file 41467_2023_42078_MOESM1_ESM.pdf]

Transcriptional repression by a secondary DNA binding surface of DNA topoisomerase I safeguards against hypertranscription

Mei Sheng Lau*, Zhenhua Hu, Xiaodan Zhao, Yaw Sing Tan, Jinyue Liu, Hua Huang, Clarisse Jingyi Yeo, Hwei Fen Leong, Oleg V. Grinchuk, Justin Kaixuan Chan, Jie Yan*, Wee-Wei Tee*

* Correspondence: M.S.L (mslau@imcb.a-star.edu.sg), J.Y (phyyj@nus.edu.sg), W-W.T (wwtee@imcb.a-star.edu.sg)

Supplementary information file contains:
Supplementary Fig. 1-11

a

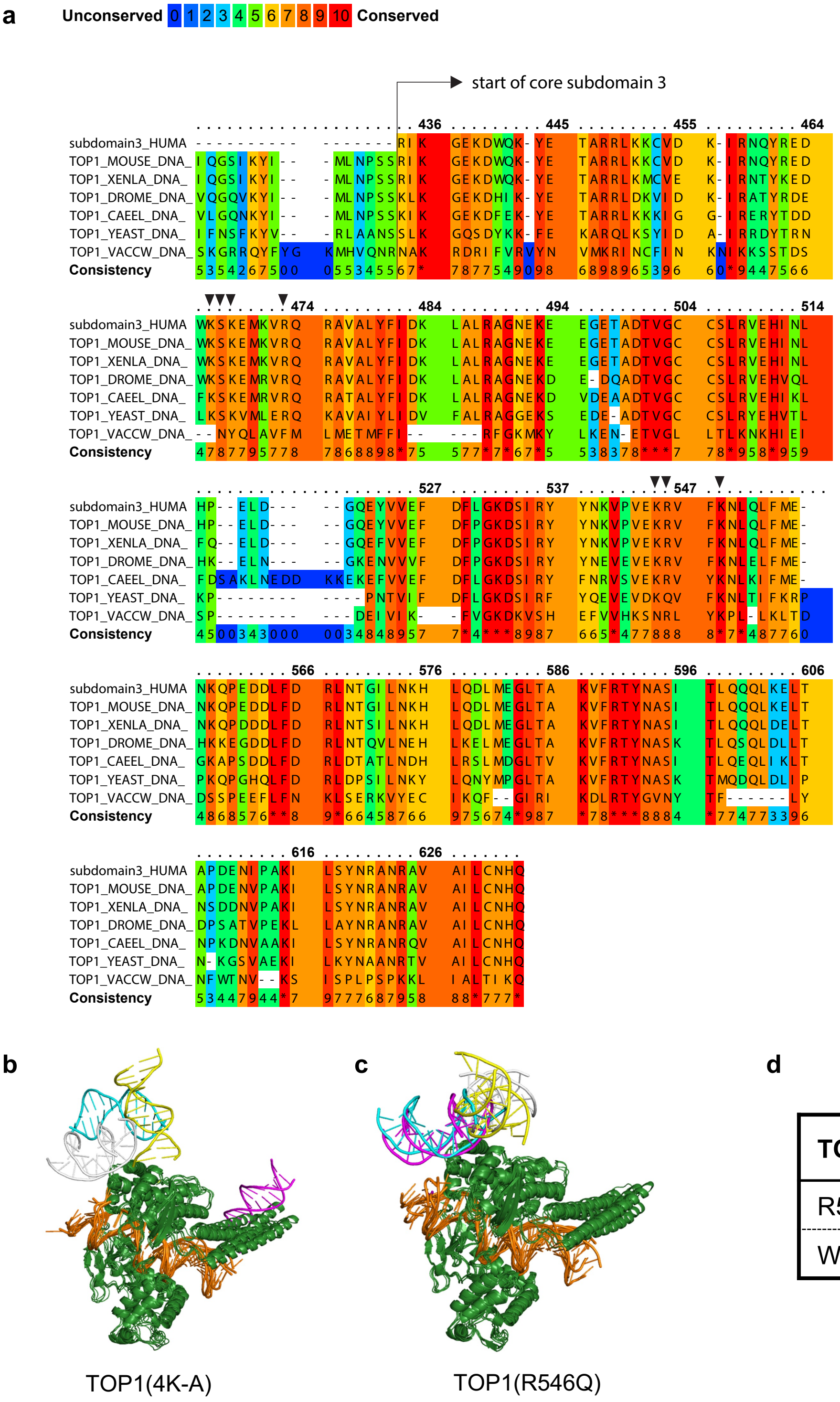

d

TOP1	ΔG(kcal/mol)
R546Q	-3.18 ± 1.97
Wildtype	-8.99 ± 2.43

Supplementary Fig. 1 DNA-interacting residues on secondary DNA binding surface are conserved across type1B topoisomerase across species and are predicted to disrupt DNA binding when mutated. (**a**) Protein sequence alignment of core subdomain III of human TOP1 (amino acids 434-633) with homologous regions of TOP1 from human, mouse, *Xenopus laevis, Drosophila melanogaster, Caenorhabditis elegans, Saccharomyces cerevisiae, and Vaccinia* virus (strain Western Reserve). Amino acid residues interacting with secondary DNA are marked with black arrow heads. Amino acid numbering is for human TOP1. (**b-c**) Superimposition of final MD trajectory structures of TOP1(4K-A)cc (**b**) and TOP1(R546Q)cc (**c**) (TOP1 in green, DNA in orange) in complex with DNA at secondary binding site from four replicate runs (yellow, magenta, cyan and white). (**d**) Computed binding free energies for the interaction of TOP1cc with secondary DNA. Values are averages ± SD obtained from the runs (two for R546Q, four for wildtype) in which secondary DNA remained stably associated with TOP1.

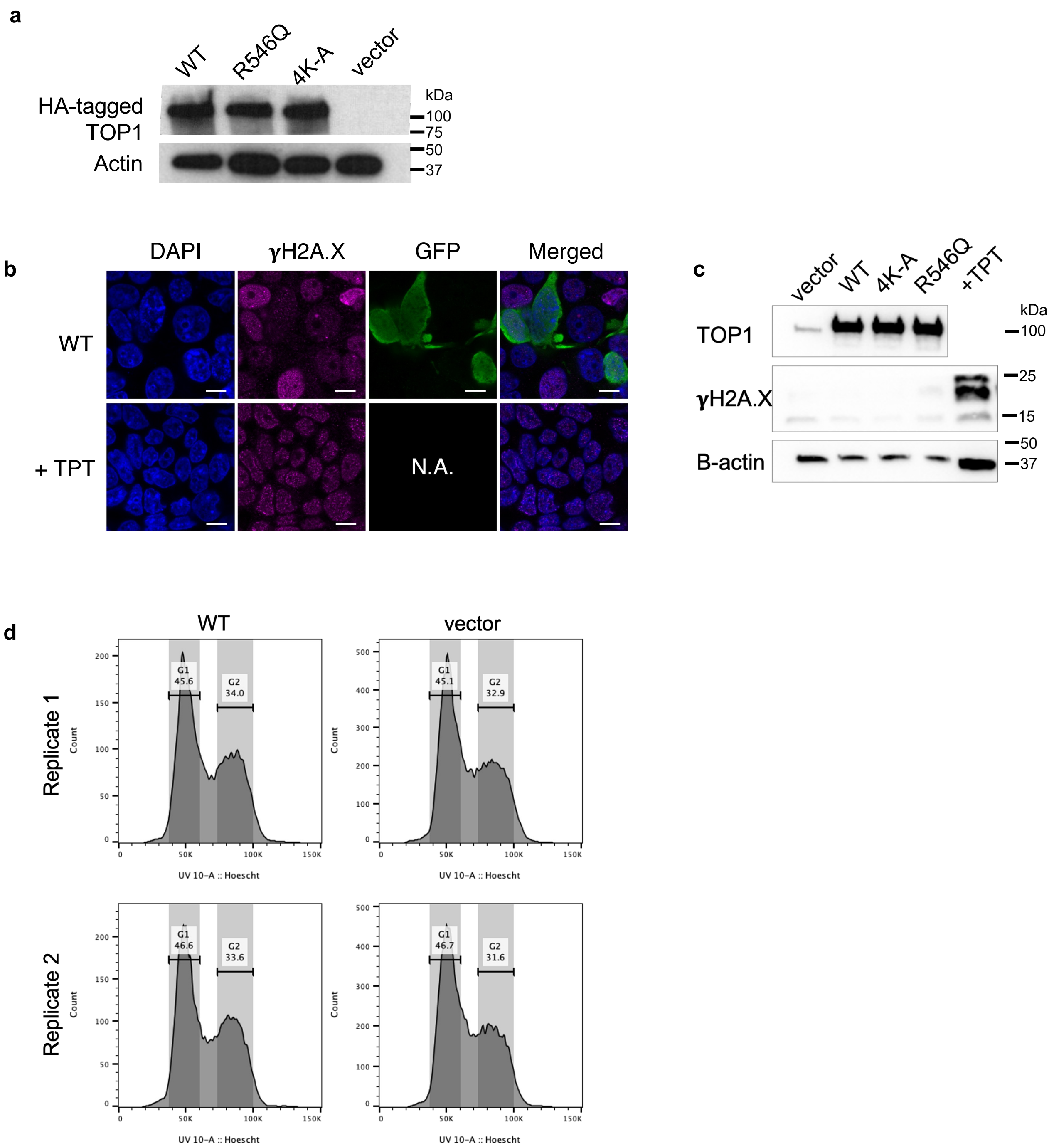

Figure S2 Overexpression of TOP1(WT), TOP1(R546Q) and TOP1(4K-A) in 293T cells. (**a**) Western blot showing protein level of exogenous HA-tagged TOP1 in lysates from equal number of sorted cells. The experiment is repeated at least twice with similar results. (**b**) Immunofluorescence images of TOP1(WT)-overexpressing cells stained for γH2A.X. GFP signal marks positively transfected cells. '+TPT' are cells treated with 100 nM topotecan (TPT) for 24 hours and is used as positive control for punctate γH2A.X signal that characterize DNA damage foci in genome. Scale bars: 10 µm. (**c**) Western blot showing total TOP1 protein levels and γH2A.X levels in lysates from cells transfected with indicated overexpression constructs. '+TPT' is lysate from cells treated with TPT as indicated in (**b**). Panels (**b**) and (**c**) are from independent experiments using orthogonal assays for determining DNA damage in cells with TOP1(WT) overexpression. (**d**) Live cell cycle analyses of TOP1(WT)-overexpressing cells and control cells.

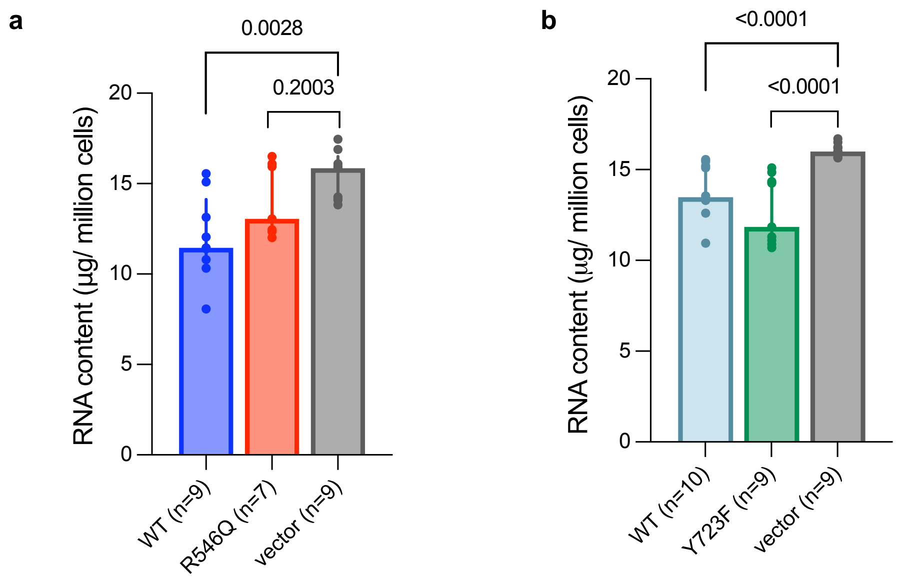

Supplementary Fig. 3 Downregulation of RNA by TOP1 is dependent on amino acid residue on secondary DNA binding site. RNA content of 293T cells overexpressing TOP1 variant proteins with mutation on secondary DNA binding surface (**a**), or mutation of catalytic residue (**b**), in comparison with TOP1(WT) and vector control. Bar heights are median and error bars are interquartile range. n, number of biological replicates, each containing 100,000 sorted cells. Mann-Whitney test was performed; *p*-values (two-tailed) are indicated above the comparisons. Source data are provided as a Source Data file.

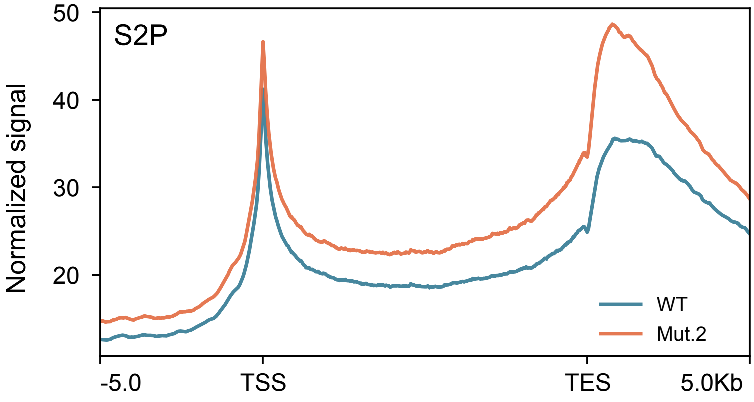

Supplementary Fig. 4 Increased transcription in mutant mESCs.
Metaplots of elongating RNAP2 (‘S2P’) ChIP-seq signal over all genes in Mut.2 and WT mESCs.

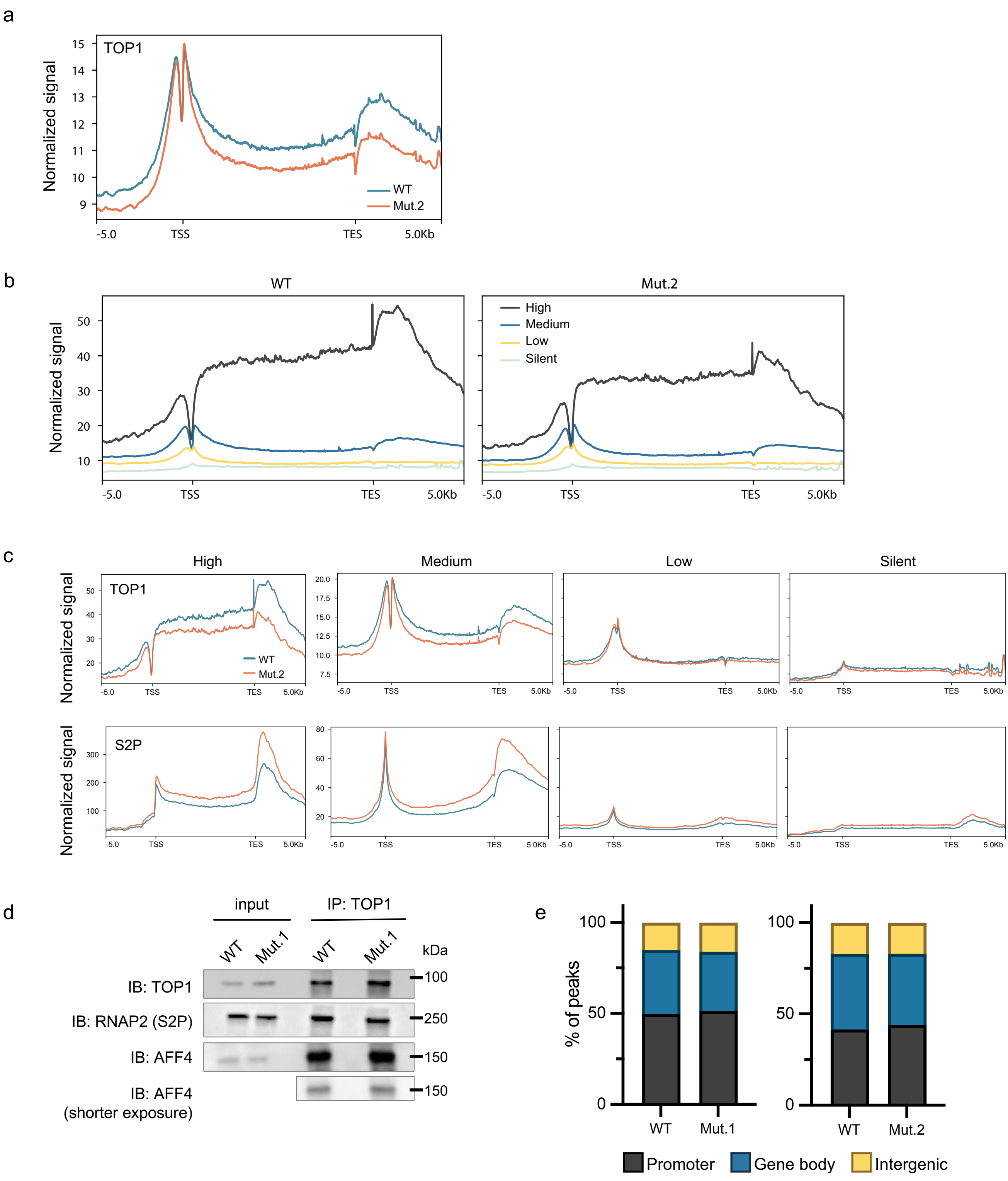

Supplementary Fig. 5 Mutant and WT TOP1 occupancy on chromatin.
(**a-b**) Metaplot of TOP1 ChIP-seq signals over all genes (**a**), or genes classified according to gene expression levels (**b**). (**c**) Metaplots of TOP1 and elongating RNAP2 ('S2P) ChIP-seq signal of over genes classified according to expression levels. Note the difference in y-axis for 'High' genes compared to the other categories. (**d**) Western blots of TOP1 co-immunoprecipitation from WT and Mut.1 mESC nuclear lysates. AFF4 is a component of the P-TEFb and Super Elongation Complex (SEC) complexes. IP: immunoprecipitated; IB: immunoblot. The experiment is repeated twice with similar results. (**e**) Distribution of TOP1 ChIP-seq peaks in various annotated regions of the genomes.

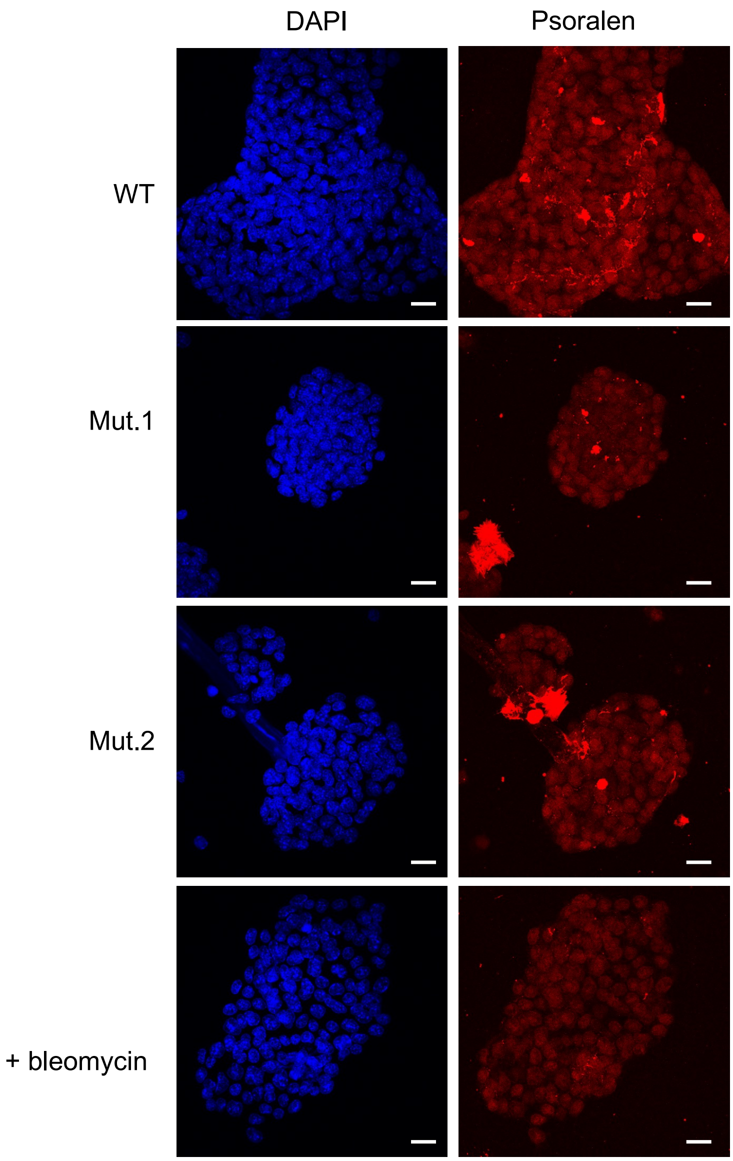

Supplementary Fig. 6 Reduced negative supercoiling in genomes of mutant mESCs.
Representative immunofluorescence images of WT and mutant mESCs with psoralen staining. Bleomycin-treated cells serves as negative control for psoralen staining. Scale bars: 20 µm. Quantification of signal in the images are presented in Fig. 3e. The experiment is repeated twice with similar results.

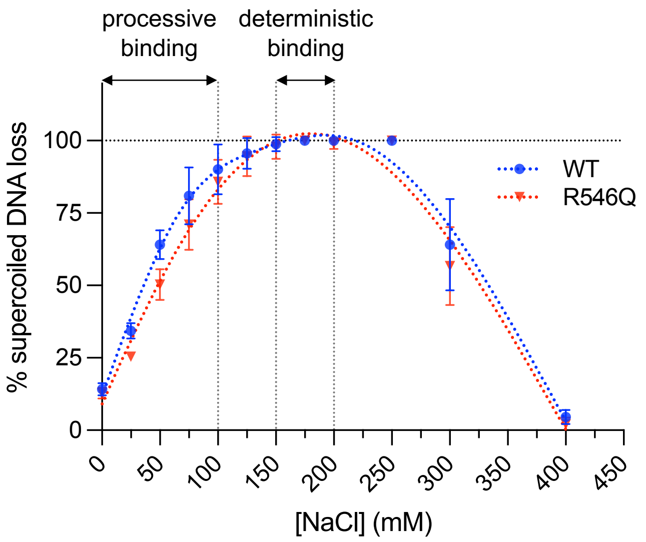

Supplementary Fig. 7 Plasmid relaxation activities of TOP1(WT) or TOP1(R546Q) recombinant proteins in varying salt conditions. n= 4 independent experiments, mean ± SD. Dotted lines are restricted cubic spline curves generated by calculating 52 points with X values ranging from 0 to 400. Source data are provided as a Source Data file.

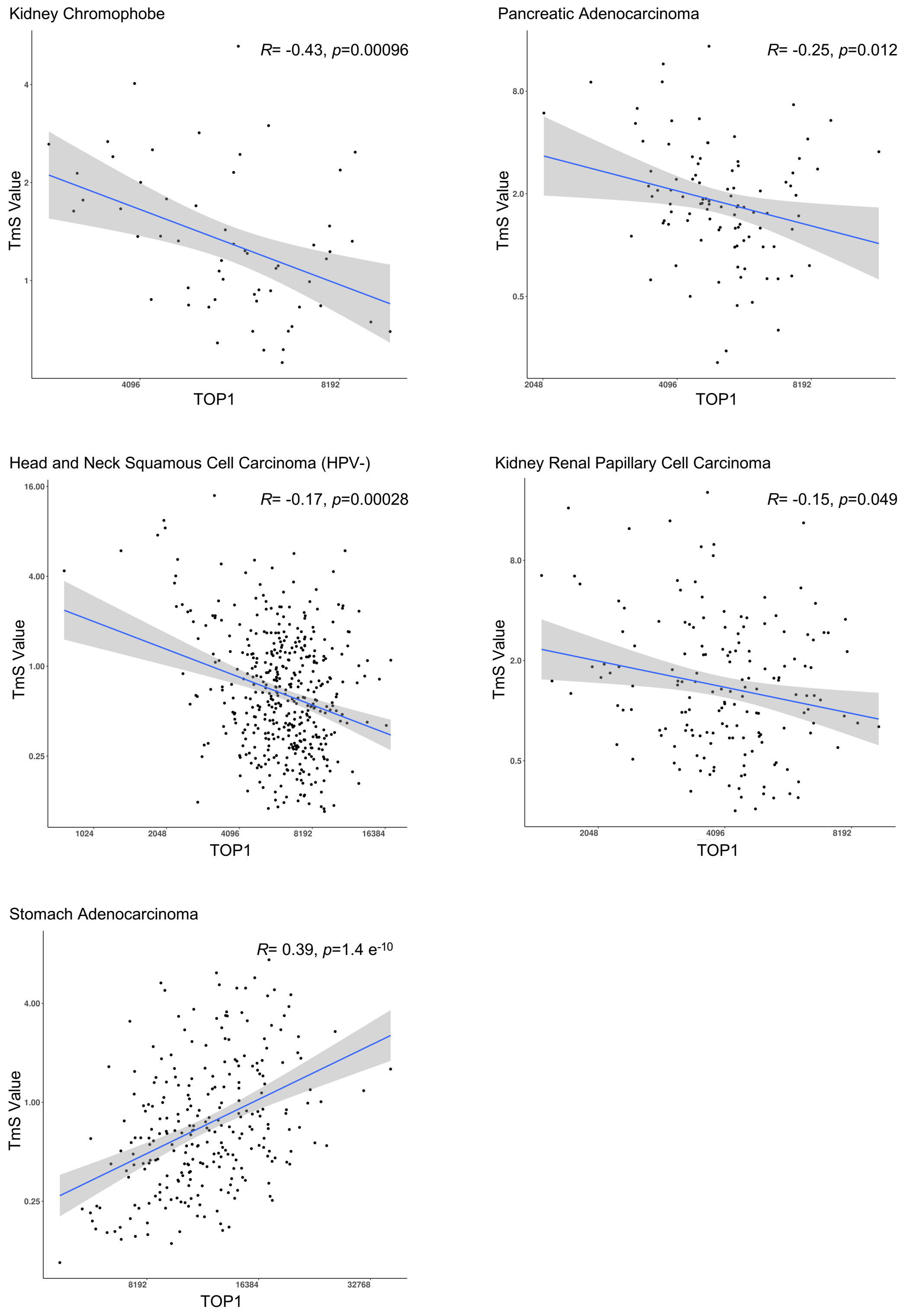

Supplementary Fig. 8 Spearman correlation plots between tumor-specific mRNA expression (TmS) and *TOP1* expression levels in indicated cancer types. Plots were generated at https://wwylab.github.io/TmS/articles/shinyapp.html using data from The Cancer Genome Atlas (TCGA) program.

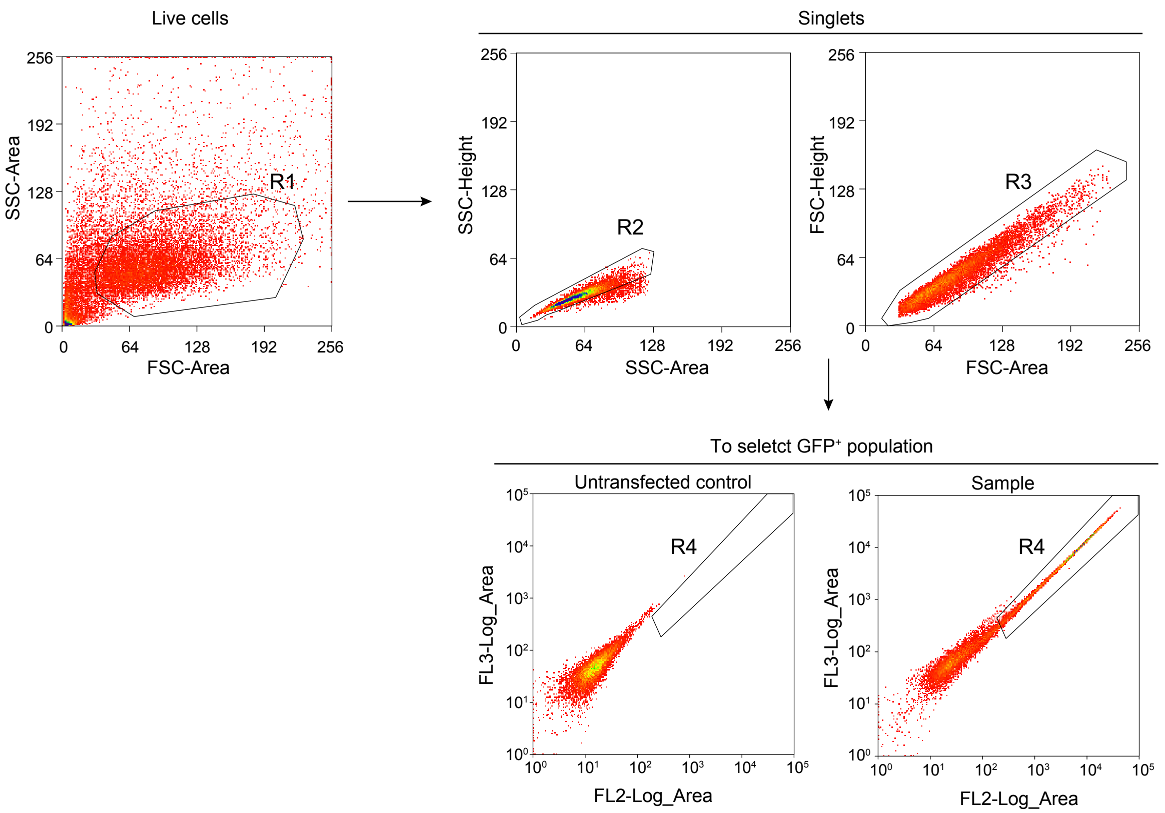

Supplementary Fig. 9 Gating strategy on flow cytometry to sort for transfected 293T cells.
The experiment was performed on Beckman-Coulter Mo-Flo Legacy Cell Sorter. FL2 and FL3 are blue lasers.

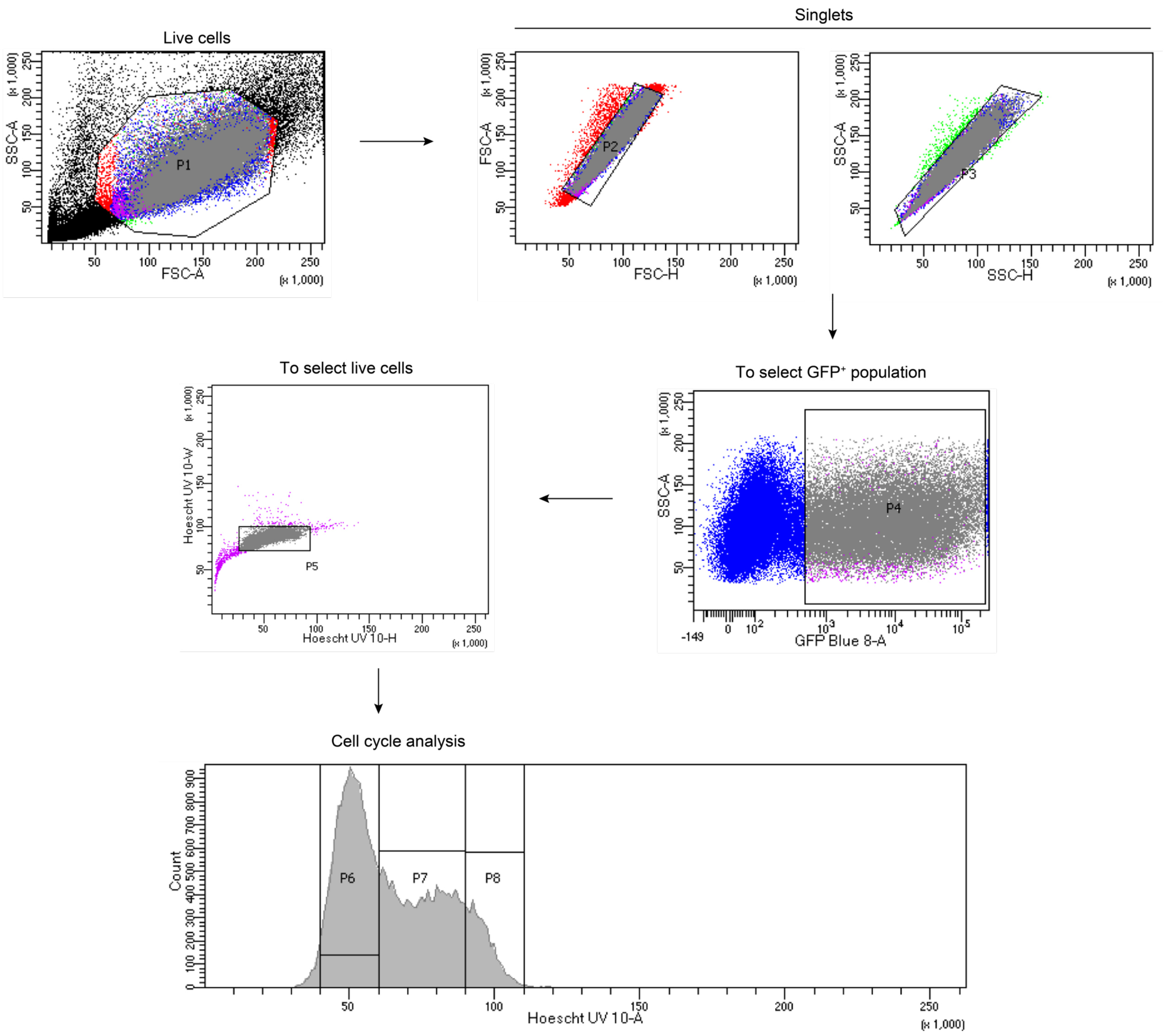

Supplementary Fig. 10 Gating strategy for cell cycle analysis of transfected 293T cells.
The experiment was performed on BD Symphony A5 cell analyzer.

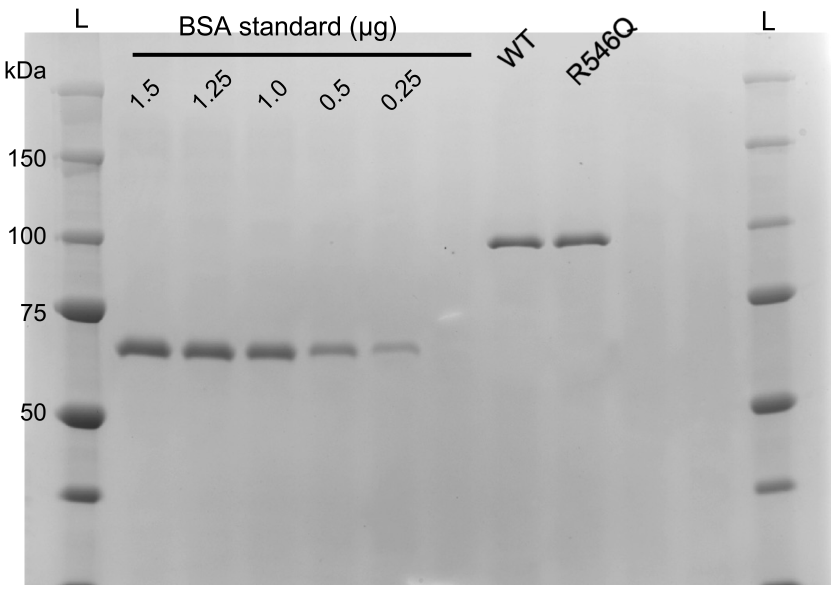

Supplementary Fig. 11 Purified recombinant TOP1(WT) and TOP1(R546Q) proteins. Coomassie blue staining of recombinant proteins and BSA standard.
