# Supplementary material for: Transcriptional repression by a secondary DNA binding surface of DNA topoisomerase I safeguards against hypertranscription
Source: Nat Commun. 2023 Oct 13;14:6464. doi: 10.1038/s41467-023-42078-9 (PMC10576097; doi:10.1038/s41467-023-42078-9)
Supplement: Supplementary file 2 — Description of Additional Supplementary Files [file 41467_2023_42078_MOESM2_ESM.docx]

**Description of Additional Supplementary Files**

**Supplementary Data 1**

Results of Gene Set Enrichment Analysis (GSEA) performed on RNA-seq comparing cells overexpressing indicated TOP1 variant and vector control.

**Supplementary Data 2**

Information on primers and DNA oligos used in this study.

**Supplementary Data 3**

Information on antibodies used in this study.
