# Supplementary material for: Transcriptional repression by a secondary DNA binding surface of DNA topoisomerase I safeguards against hypertranscription
Source: Nat Commun. 2023 Oct 13;14:6464. doi: 10.1038/s41467-023-42078-9 (PMC10576097; doi:10.1038/s41467-023-42078-9)
Supplement: Supplementary file 6 — Reporting Summary [file 41467_2023_42078_MOESM6_ESM.pdf]

nature portfolio

Corresponding author(s): Mei Sheng Lau, Jie Yan, Wee-Wei Tee

Last updated by author(s): 2023/09/05

Reporting Summary

Nature Portfolio wishes to improve the reproducibility of the work that we publish. This form provides structure for consistency and transparency in reporting. For further information on Nature Portfolio policies, see our Editorial Policies and the Editorial Policy Checklist.

Statistics

For all statistical analyses, confirm that the following items are present in the figure legend, table legend, main text, or Methods section.

n/a	Confirmed	
☐	☒	The exact sample size (*n*) for each experimental group/condition, given as a discrete number and unit of measurement
☐	☒	A statement on whether measurements were taken from distinct samples or whether the same sample was measured repeatedly
☐	☒	The statistical test(s) used AND whether they are one- or two-sided<br>*Only common tests should be described solely by name; describe more complex techniques in the Methods section.*
☒	☐	A description of all covariates tested
☐	☒	A description of any assumptions or corrections, such as tests of normality and adjustment for multiple comparisons
☐	☒	A full description of the statistical parameters including central tendency (e.g. means) or other basic estimates (e.g. regression coefficient) AND variation (e.g. standard deviation) or associated estimates of uncertainty (e.g. confidence intervals)
☐	☒	For null hypothesis testing, the test statistic (e.g. *F*, *t*, *r*) with confidence intervals, effect sizes, degrees of freedom and *P* value noted<br>*Give P values as exact values whenever suitable.*
☒	☐	For Bayesian analysis, information on the choice of priors and Markov chain Monte Carlo settings
☒	☐	For hierarchical and complex designs, identification of the appropriate level for tests and full reporting of outcomes
☒	☐	Estimates of effect sizes (e.g. Cohen's *d*, Pearson's *r*), indicating how they were calculated

Our web collection on statistics for biologists contains articles on many of the points above.

Software and code

Policy information about availability of computer code

Data collection	For molecular dynamic (MD) simulation- AMBER 18;<br>For structural modelling - PyMOL (version 1.3), PDB2PQR (version 1. 7);<br>For loop modelling- ModLoop web server (accessed on 6 Aug 2021);<br>For cell sorting- Summit (version 5.4) on Mo-Flo;<br>For flow cytometry- FACSDiva (version 9.8) on BD Symphony AS analyzer;<br>For microscopy imaging- FV31S-SW (version 2.4.l.198), Zen Blue (version 3.3.89.0000);<br>For RNA-seq, ChIP-seq and PRO-seq- Illumina HiSeq-2000 or Novaseq platforms;<br>For DNA gel image acquisition- Gel Doc XR+ (version 5.2);<br>For magnetic tweezer experiments - LabVIEW 2015;<br>For patch clamp- Clampex (version 10.7.0.3).
Data analysis	For analysis of MD simulation- AMBER 18;<br>For visualization of structures and generation of figures- PyMOL version 1.3;<br>For visualization of MD simulations- VMD version 1.9.2.<br><br>For analysis of sequencing data, RNA-seq and ChIP-seq-<br>Trim Galore (version 0.4.2_dev); RSEM(version 1.1.11); DESeq2 (version 1.16.1) ; Bowtie2(version .2.2.9); SAMtools(version 1.4); deeptools (version 2.5.3); R (version 4.0.5); Integrative Genomics Viewer (IGV 2.10.2).<br><br>For PRO-seq analysis-<br>UMI-tools (version 1.1.4; DOI:10.1101/gr.209601.116); cutadapt (version 1.14; DOI:10.14806/ej.17.l.200); seqtk trimfq (version 1.3-r119-

dirty; https://github.com/lh3/seqtk); bowtie2stdBedGraph.pl (version 1 https://doi.org/10.5281/zenodo.5519915); proTSScall (version 1; https://doi.org/10.5281/zenodo.8298661), make_heatmap (version 1 https://doi.org/10.5281/zenodo.5519915).

For protein sequence alignment- PRALINE (https://www.ibi.vu.nl/programs/pralinewww/).
For cloning over-expression constructs- Benchling (https:// www.benchling.com).
For statistical analysis - Jupyter (6.5.4), Prism (9.5.0).
For image analyses- Imaris (version 9.2).
For cell cycle analysis- FlowJo (versino 10.8.2).
For DNA gel analysis- Image Lab (version 6.1).
For patch clamp analysis - pCLAMP (version 10.5)

For manuscripts utilizing custom algorithms or software that are central to the research but not yet described in published literature, software must be made available to editors and reviewers. We strongly encourage code deposition in a community repository (e.g. GitHub). See the Nature Portfolio guidelines for submitting code & software for further information.

Data

Policy information about availability of data

All manuscripts must include a data availability statement. This statement should provide the following information, where applicable:

- Accession codes, unique identifiers, or web links for publicly available datasets
- A description of any restrictions on data availability
- For clinical datasets or third party data, please ensure that the statement adheres to our policy

The MD simulation input files and processed output trajectories have been deposited in Zenodo and are available at https://doi.org/10.5281/zenodo.8158854. The raw and processed sequencing data generated in this study have been deposited in the NCBI Gene Expression Omnibus (GEO) under accession number GSE207163 [https://www.ncbi.nlm.nih.gov/geo/query/acc.cgi?acc=GSE207163]. The crystal structures PDB code 1A36 and PDB code 3M4A were retrieved from Protein Data Bank [www.wwpdb.org]. Human hg19, mouse mm10 and fly dm6 reference genomes was obtained from GENCODE [https://www.gencodegenes.org/]. Molecular Signatures Database (MSigDB) was obtained from https://www.gsea-msigdb.org/gsea/index.jsp. All other data are available in the main article, Supplementary Information, and source data. Source data are provided with this paper.

Research involving human participants, their data, or biological material

Policy information about studies with human participants or human data. See also policy information about sex, gender (identity/presentation), and sexual orientation and race, ethnicity and racism.

Reporting on sex and gender	N/A
Reporting on race, ethnicity, or other socially relevant groupings	N/A
Population characteristics	N/A
Recruitment	N/A
Ethics oversight	N/A

Note that full information on the approval of the study protocol must also be provided in the manuscript.

Field-specific reporting

Please select the one below that is the best fit for your research. If you are not sure, read the appropriate sections before making your selection.

☒ Life sciences ☐ Behavioural & social sciences ☐ Ecological, evolutionary & environmental sciences

For a reference copy of the document with all sections, see nature.com/documents/nr-reporting-summary-flat.pdf

Life sciences study design

All studies must disclose on these points even when the disclosure is negative.

Sample size	Sample sizes for each experiments are provided in the figures and corresponding figure legends. Samples sizes were determined based on common practices in the field. For example, see PMID: 37117180, PMID:28591571, PMID 34301855, PMID: 20164500, PMID:35914168.
Data exclusions	No data were excluded from the experiments.
Replication	All experiments are independently repeated two to three times. The observed biological effects of interest were consistent between replicates. Key conclusions in the paper were further replicated using independent methods, and through using additional cell line.

Randomization	No randomization was performed because no subjective process was involved in data collection and analyses.
Blinding	Blinding were not applicable because the results collected are quantitative in nature (i.e. not subjective), and samples for comparison were collected and analyzed under the same conditions.

Reporting for specific materials, systems and methods

We require information from authors about some types of materials, experimental systems and methods used in many studies. Here, indicate whether each material, system or method listed is relevant to your study. If you are not sure if a list item applies to your research, read the appropriate section before selecting a response.

Materials & experimental systems

n/a	Involved in the study	
☐	☒	Antibodies
☐	☒	Eukaryotic cell lines
☒	☐	Palaeontology and archaeology
☒	☐	Animals and other organisms
☒	☐	Clinical data
☒	☐	Dual use research of concern
☒	☐	Plants

Methods

n/a	Involved in the study	
☐	☒	ChIP-seq
☐	☒	Flow cytometry
☒	☐	MRI-based neuroimaging

Antibodies

Antibodies used

Antibody, Manufacturer, Catalog number
Rabbit polyclonal anti-TOPI antibody, Bethyl, A302-590A
Rabbit polyclonal anti-RNA polymerase II CTD repeat YSPTSPS (PhosphoS2), Abcam, ab5095
Mouse monoclonal anti-HA.11 epitope Tag antibody, Biolegend, 901501
Mouse monoclonal anti-actin (C-4),Santa-Cruz, sc-47778
Rabbit polyclonal anti-AFF4, Proteintech, 14662-1-AP
Rabbit polyclonal anti-gamma H2A.X (phospho S139), Abcam, ab2893
Goat polyclonal anti-GFP, Abcam, ab6673
Alexa Fluor 594 Streptavidin, Thermofisher, S32356
Goat anti-Rabbit IgG HRP-Conjugated, Bethyl, A120-201P
Goat anti-Mouse IgG HRP-Conjugated, Bethyl, A90-516P
Donkey anti-Goat IgG (H+L) Secondary Antibody, Alexa Fluor 488 conjugate, Thermofisher, A11055
Donkey anti-Rabbit IgG (H+L) Secondary Antibody, Alexa Fluor 594 conjugate, Thermofisher, A21207

Validation

Rabbit polyclonal anti-TOPI antibody, Bethyl, A302-590A
This antibody is validated by manufacturer for immunoprecipitation and Western blot applications.
The specificity of this antibody was further validated by us through Western blot comparing cell samples that over-expressed human TOP1 with control samples (Supplementary Fig. 2c), and mESCs treated with siRNAs against Top1 and non-targeting siRNAs (data not shown).

Rabbit polyclonal anti-RNA polymerase II CTD repeat YSPTSPS (PhosphoS2), Abcam, ab5095
This antibody has been used for ChIP in mouse samples in multiple publications (e.g. PubMed: 25735743, PubMed: 28234895). It is validated for Western blot by manufacturer.

Mouse monoclonal anti-HA.11 epitope Tag antibody, Biolegend, 901501
The specificity of this antibody is validated by us through Western blot comparing cell samples that over-expressed HA-tagged TOP1 with control (Supplementary Fig. 2a).

Mouse monoclonal anti-actin (C-4),Santa-Cruz, sc-47778
This is a widely used antibody (with > 13k citations). This publication PMID: 2460261 reports the specificity of this clone against vertebrate actin.

Rabbit polyclonal anti-AFF4, Proteintech, 14662-1-AP
This antibody was validated by others for Western blot by using knockdown experiments in their publication (PMID: 27353326).

Rabbit polyclonal anti-gamma H2A.X (phospho S139), Abcam, ab2893
This antibody was validated by manufacturer for Western blot and immunofluorescence imaging by comparing samples treated with and without DNA damaging agents.

Goat polyclonal anti-GFP, Abcam, ab6673
This antibody was validated by manufacturer for IF through staining of transgenic mouse tissues with tissue-specific GFP expression.

Eukaryotic cell lines

Policy information about cell lines and Sex and Gender in Research

Cell line source(s)	293T cells and E14 mouse embryonic stem cells were obtained from ATCC.
Authentication	293T cells were not authenticated by ATCC through STR profiling. E14 cells verified with based on morphology and pluripotent gene expression profile.
Mycoplasma contamination	Cells were tested routinely for mycoplasm and are free of contamination at the point of our experiments.
Commonly misidentified lines (See ICLAC register)	No commonly misidentified cell lines were used in the study.

Plants

Seed stocks	N/A
Novel plant genotypes	N/A
Authentication	N/A

ChIP-seq

Data deposition

☒ Confirm that both raw and final processed data have been deposited in a public database such as GEO.

☒ Confirm that you have deposited or provided access to graph files (e.g. BED files) for the called peaks.

Data access links
May remain private before publication.

Raw and processed sequencing data generated in this study have been deposited in the NCBI Gene Expression Omnibus (GEO) under accession number GSE207163 [https://www.ncbi.nlm.nih.gov/geo/query/acc.cgi?acc=GSE207163].

Files in database submission

ChIP-seq.mESC.RNAP2S2P_Mut.1_rep12.bw
ChIP-seq.mESC.RNAP2S2P_Mut.2_rep1.bw
ChIP-seq.mESC.RNAP2S2P_WT_rep12.bw
ChIP-seq.mESC.RNAP2S2P_WT_rep1.bw
ChIP-seq.mESC.TOP1_Mut.1_rep123.bw
ChIP-seq.mESC.TOP1_Mut.2.bw
ChIP-seq.mESC.TOP1_WT-matched-to-Mut.1_rep123.bw
ChIP-seq.mESC.TOP1_WT-matched-to-Mut.2.bw
ChIP-seq.mESC.TOP1_Mut.1_rep123.bed
ChIP-seq.mESC.TOP1_Mut.2.bed
ChIP-seq.mESC.TOP1_WT-matched-to-Mut.1_rep123.bed
ChIP-seq.mESC.TOP1_WT-matched-to-Mut.2.bed
ChIP-seq.mESC.RNAP2S2P_Mut.1_rep1_input_1.fastq.gz
ChIP-seq.mESC.RNAP2S2P_Mut.1_rep1_input_2.fastq.gz
ChIP-seq.mESC.RNAP2S2P_Mut.1_rep1_IP_1.fastq.gz
ChIP-seq.mESC.RNAP2S2P_Mut.1_rep1_IP_2.fastq.gz
ChIP-seq.mESC.RNAP2S2P_Mut.1_rep2_input_1.fastq.gz
ChIP-seq.mESC.RNAP2S2P_Mut.1_rep2_input_2.fastq.gz
ChIP-seq.mESC.RNAP2S2P_Mut.1_rep2_IP_1.fastq.gz
ChIP-seq.mESC.RNAP2S2P_Mut.1_rep2_IP_2.fastq.gz
ChIP-seq.mESC.RNAP2S2P_Mut.2_rep1_input_1.fastq.gz
ChIP-seq.mESC.RNAP2S2P_Mut.2_rep1_input_2.fastq.gz
ChIP-seq.mESC.RNAP2S2P_Mut.2_rep1_IP_1.fastq.gz
ChIP-seq.mESC.RNAP2S2P_Mut.2_rep1_IP_2.fastq.gz
ChIP-seq.mESC.RNAP2S2P_WT_rep1_input_1.fastq.gz
ChIP-seq.mESC.RNAP2S2P_WT_rep1_input_2.fastq.gz
ChIP-seq.mESC.RNAP2S2P_WT_rep1_IP_1.fastq.gz
ChIP-seq.mESC.RNAP2S2P_WT_rep1_IP_2.fastq.gz
ChIP-seq.mESC.RNAP2S2P_WT_rep2_input_1.fastq.gz
ChIP-seq.mESC.RNAP2S2P_WT_rep2_input_2.fastq.gz
ChIP-seq.mESC.RNAP2S2P_WT_rep2_IP_1.fastq.gz
ChIP-seq.mESC.RNAP2S2P_WT_rep2_IP_2.fastq.gz
ChIP-seq.mESC.TOP1_Mut.1_rep1_input.fastq.gz
ChIP-seq.mESC.TOP1_Mut.1_rep1_IP.fastq.gz
ChIP-seq.mESC.TOP1_Mut.1_rep2_input.fastq.gz
ChIP-seq.mESC.TOP1_Mut.1_rep2_IP.fastq.gz
ChIP-seq.mESC.TOP1_Mut.1_rep3_input.fastq.gz
ChIP-seq.mESC.TOP1_Mut.1_rep3_IP.fastq.gz
ChIP-seq.mESC.TOP1_Mut.2_input_1.fastq.gz

ChIP-seq.mESC.TOP1_Mut.2_input_2.fastq.gz
ChIP-seq.mESC.TOP1_Mut.2_IP_1.fastq.gz
ChIP-seq.mESC.TOP1_Mut.2_IP_2.fastq.gz
ChIP-seq.mESC.TOP1_WT-matched-to-Mut.1_rep1_input.fastq.gz
ChIP-seq.mESC.TOP1_WT-matched-to-Mut.1_rep1_IP.fastq.gz
ChIP-seq.mESC.TOP1_WT-matched-to-Mut.1_rep2_input.fastq.gz
ChIP-seq.mESC.TOP1_WT-matched-to-Mut.1_rep2_IP.fastq.gz
ChIP-seq.mESC.TOP1_WT-matched-to-Mut.1_rep3_input.fastq.gz
ChIP-seq.mESC.TOP1_WT-matched-to-Mut.1_rep3_IP.fastq.gz
ChIP-seq.mESC.TOP1_WT-matched-to-Mut.2_input_1.fastq.gz
ChIP-seq.mESC.TOP1_WT-matched-to-Mut.2_input_2.fastq.gz
ChIP-seq.mESC.TOP1_WT-matched-to-Mut.2_IP_1.fastq.gz
ChIP-seq.mESC.TOP1_WT-matched-to-Mut.2_IP_2.fastq.gz

Genome browser session
(e.g. UCSC)

N/A

Methodology

Replicates

Mut.1 and Mut.2 are independent mutant clones. ChIP experiments with Mut.2 are for replicating findings in Mut.1 in an independent cell line.
For RNAP2(S2P) ChIP, there are two biological replicates for Mut.1, one replicate for Mut.2, and two biological replicates for WT. For TOP1 ChIP, there are three biological replicates for the Mut.1 and WT pair, and one replicate for the Mut.2 and WT pair.

Sequencing depth

sample total# of reads uniquely mapped reads read length sequencing type
ChIP-seq.mESC.RNAP2S2P_Mut.1_rep1_input 49,757,885 35,453,333150bp paired-end
ChIP-seq.mESC.RNAP2S2P_Mut.1_rep1_lP 64,322,863 48,473,303 150bp paired-end
ChIP-seq.mESC.RNAP2S2P_Mut.1_rep2_input 53,598,012 38,433,693 150bp paired-end
ChIP-seq.mESC.RNAP2S2P_Mut.1_rep2_IP 66,250,432 50,979,552 150bp paired-end
ChIP-seq.mESC.RNAP2S2P_Mut.2_rep1_input 53,914,652 39,140,343 150bp paired-end
ChIP-seq.mESC.RNAP2S2P_Mut.2_rep1_lP 73,820,340 54,628,511150bp paired-end
ChIP-seq.mESC.RNAP2S2P_WT_rep1_input 47,390,329 34,306,104150bp paired-end
ChIP-seq.mESC.RNAP2S2P_WT_rep1_lP 84,113,446 63,657,088150bp paired-end
ChIP-seq.mESC.RNAP2S2P_WT_rep2_input 47,782,401 34,531,734 150bp paired-end
ChIP-seq.mESC.RNAP2S2P_WT_rep2_IP 66,888,225 51,304,996150bp paired-end
ChIP-seq.mESC.TOP1_Mut.2_input 50,344,436 36,974,396150bp paired-end
ChIP-seq.mESC.TOP1_Mut.2_IP 55,659,686 38,841,328 150bp paired-end
ChIP-seq.mESC.TOP1_WT-matched-to-Mut.2_input 56,808,380 42,294,678 150bp paired-end
ChIP-seq.mESC.TOP1_WT-matched-to-Mut.2_IP 50,192,540 35,275,855 150bp paired-end
ChIP-seq.mESC.TOP1_Mut.1_rep1_input 79,252,655 50,315,362 76bp single-end
ChIP-seq.mESC.TOP1_Mut.1_rep1_lP 73,619,369 43,297,493 76bp single-end
ChIP-seq.mESC.TOP1_WT-matched-to-Mut.1_rep1_input 81,714,705 55,145,784 76bp single-end
ChIP-seq.mESC.TOP1_WT-matched-to-Mut.1_rep1_lP 65,225,128 41,843,131 76bp single-end
ChIP-seq.mESC.TOP1_Mut.1_rep2_input 52,522,240 35,976,221 76bp single-end
ChIP-seq.mESC.TOP1_Mut.1_rep2_IP 44,262,41129,009,765 76bp single-end
ChIP-seq.mESC.TOP1_Mut.1_rep3_input 44,514,503 30,909,368 76bp single-end
ChIP-seq.mESC.TOP1_Mut.1_rep3_IP 57,141,709 37,134,006 76bp single-end
ChIP-seq.mESC.TOP1_WT-matched-to-Mut.1_rep2_input 47,427,267 32,684,962 76bp single-end
ChIP-seq.mESC.TOP1_WT-matched-to-Mut.1_rep2_IP 52,938,465 34,462,037 76bp single-end
ChIP-seq.mESC.TOP1_WT-matched-to-Mut.1_rep3_input 51,118,38135,382,877 76bp single-end
ChIP-seq.mESC.TOP1_WT-matched-to-Mut.1_rep3_IP 47,682,826 30,868,142 76bp single-end

Antibodies

Rabbit polyclonal anti-TOPl antibody, Bethyl, A302-590A
Rabbit polyclonal anti-RNA polymerase II CTD repeat YSPTSPS (PhosphoS2), Abcam, ab5095

Peak calling parameters

For single-end TOP1 ChIP dataset:
macs2 callpeak-t IP.rmdup.bam -c INPUT.rmdup.bam -f BAMPE -g mm --broad

For paired-end TOP1 ChIP dataset:
macs2 callpeak-t IP.rmdup.bam -c INPUT.rmdup.bam -f BAM -g mm --broad --keep-dup all
"keep-dup all" is used because the PCR duplicates have already been removed earlier in the pipeline.

Data quality

Only unique reads with MAPQ >= 10 were kept; PCR duplicates and reads falling in blacklist regions were filtered out.

Software

Trim Galore (v0.4.2_dev); Bowtie2(v.2.2.9); SAMtools(vl.4); deeptools (v2.5.3); macs2(v2.1.1)

Flow Cytometry

Plots

Confirm that:

☒ The axis labels state the marker and fluorochrome used (e.g. CD4-FITC).

☒ The axis scales are clearly visible. Include numbers along axes only for bottom left plot of group (a 'group' is an analysis of identical markers).

☒ All plots are contour plots with outliers or pseudocolor plots.

☐ A numerical value for number of cells or percentage (with statistics) is provided.

Methodology

Sample preparation	For RNA-seq and RNA content determination, transfected 293T cells were trypsinized, resuspended in complete media supplemented with DNaseI to prevent clumping, passed through a cell strainer (0.7 um) into FACS tube, and transported on ice to sort facility. 1 ug/mL propidium iodide was added just before sorting to enable exclusion of dead cells in the sort.<br><br>For cell cycle analysis, transfected 293T cells were trypsinized and incubated in 20 ug/mL Hoescht33342 in FACS buffer (lxPBS, 5% FBS) for 20 min at 37 deg C. After one wash with PBS, cells were resuspended in FACS buffer, passed through a cell strainer (0.7 um) into FACS tube, and transported on ice to FACS facility.
Instrument	Sorting was performed on Beckman-Coulter Mo-Flo Legacy Cell Sorter; FACS was performed on BD FACSymphony A5.2.
Software	Summit (Version 5.4) for cell sorter; FACSDiva (version 9.8) for flow cytometry data collection; FlowJo (Version 10.8.2) for cell cycle analysis.
Cell population abundance	Purity was determined by re-sorting a post-sort fraction. Purity is typically greater than 95%.
Gating strategy	Gating strategies were described in Supplementary Figures 9 and 10.

☒ Tick this box to confirm that a figure exemplifying the gating strategy is provided in the Supplementary Information.
